# Development of routine-data-compatible quality indicators for the management of osteoarthritis of the knee and hip in ambulatory care: a RAND-modified Delphi consensus process

**DOI:** 10.1186/s13018-025-06127-x

**Published:** 2025-07-26

**Authors:** Tobias Bock, Philip Bammert, Ronja Flemming, Rüdiger von Eisenhart-Rothe, Igor Lazic, Leonie Sundmacher

**Affiliations:** 1 Chair of Health Economics, Department Health and Sport Sciences, TUM School of Medicine and Health, Technical University of Munich, Am Olympiacampus 11, Munich, 80809 Germany; 2 Munich Center for Health Economics and Policy, Munich, Germany; 3 Department of Orthopaedics and Sport Orthopaedics, University Hospital rechts der Isar, Technical University of Munich, Munich, Germany

## Abstract

**Objective:**

High rates of total joint arthroplasty (TJA) in patients with osteoarthritis of the knee and hip in Germany have raised concerns about the insufficient use of ambulatory health care services. Quality indicators (QIs) can help assess the provision of care and identify deficits in health care delivery. To build on a preceding systematic review, we conducted a Delphi process to develop QIs that can be quantified using routine data from German statutory health insurers and used for the ambulatory management of osteoarthritis of the knee and hip before TJA.

**Methods:**

We conducted a three-round RAND-modified Delphi consensus process consisting of an initial online questionnaire (Round 1), a virtual panel meeting with an additional online questionnaire (Round 2), and a final online questionnaire (Round 3). A panel of eight physicians, one physical therapist and one patient advocacy organisation representative evaluated QIs derived from a systematic review for relevance, clarity and feasibility. The panel also proposed additional QIs for consideration.

**Results:**

Following pre-selection by two non-panel physicians, 19 of the 24 QIs identified in the systematic review were included in the first Delphi round. During the panel meeting, panellists proposed six additional QIs. Ultimately, consensus was reached on twelve routine-data-compatible QIs related to diagnostic imaging, exercise therapy, medication, preconditions for TJA, and interprofessional collaboration among physicians.

**Conclusion:**

The evidence- and consensus-based QIs are intended to support health care providers in monitoring and improving care processes, and may serve as a basis for identifying and addressing variation in care quality.

**Supplementary Information:**

The online version contains supplementary material available at 10.1186/s13018-025-06127-x.

## Background

Osteoarthritis (OA) is a leading cause of disability and morbidity, imposing a substantial physical and psychosocial burden on affected individuals [11]. Globally, the prevalence of OA increased by 132.2% from 256 million individuals in 1990 to 595 million individuals in 2020 [14]. Given ageing populations and rising life expectancies, this prevalence is expected to increase markedly in the coming years [34]. The economic burden of OA results not only from direct medical expenses but also from considerable indirect costs, including loss of labour productivity due to work absences and early retirement [37]. Patients with OA of the knee (KOA) and hip (HOA) are among the most frequent users of health care services. In an analysis of the North Rhine region of Germany, patients with a KOA or HOA diagnosis accounted for a substantial proportion of treatment cases in orthopaedic (23.3%), physical and rehabilitative medicine (21.3%) and general medicine (8.7%) practices in the first quarter of 2024 [22].

Although total joint arthroplasty (TJA) is a highly successful surgical procedure for patients with end-stage OA – relieving pain, restoring joint function and reducing the risk of comorbidities [18, 32, 47] – the decision to proceed with surgery must be determined with particular caution, given that the resection of the articular surfaces constitutes an irreversible intervention. Guidelines therefore recommend that TJA should only be performed when ambulatory interventions have been thoroughly explored, taking into account both the patient’s individual level of subjective suffering and the severity of OA [8, 9]. In recent years, however, Germany has reported some of the highest knee and hip TJA rates internationally [33]. This has sparked ongoing debate about whether these rates reflect the needs of an ageing population or an overutilisation of health care resources. Factors such as misaligned financial incentives in the diagnosis-related group (DRG) payment system and regional variation in rates of TJA have been cited as potential contributors [25, 31, 38].

High-quality OA care should incorporate interprofessional collaboration, evidence-based interventions and quality indicators (QIs) to assess adherence to best practices. QIs provide a scientific basis for monitoring care quality and driving quality improvements [20]. However, integrating quality assurance into health care billing systems remains a challenge, as standardised and efficient methods for measuring care quality in routine practice are lacking. Routine data offer a practical solution for quantifying QIs and enabling reliable quality assessments [19]. Although QIs exist for OA care in Germany, they focus exclusively on discharge management following TJA or rely on clinical data collection [24, 43]. Currently, no routine-data-compatible QI set is available that can be used to evaluate the quality of the ambulatory care provided to patients with KOA and HOA before TJA in Germany and quantified using routine data from German statutory health insurers (SHIs). To address this research gap, we conducted a RAND-modified Delphi consensus process to develop a set of routine-data-compatible QIs based on the findings from a preceding systematic review [3].

## Materials and methods

The Delphi process reported in this paper is part of the first phase of the MobilE-ARTH study (German Clinical Trials Register, ID: DRKS00027516, Registration Date: 2022-01-25), which aims to improve the quality of pre-TJA ambulatory care for patients with KOA or HOA. The study was approved by the Ethics Committee of the Faculty of Medicine at the Technical University of Munich (566/21 S-NP, granted 2021-10-13). The three-phase study design consists of the development, pilot testing and evaluation of routine-data-compatible QIs [4].

We conducted a Delphi process based on the RAND/UCLA Appropriateness Method (RAM), which provides a more appropriate and structured framework for the systematic development of QIs in health care than formal Delphi guideline approaches. First, RAM explicitly focuses on assessing the appropriateness of clinical interventions, a central aspect when developing QIs intended to detect potential overuse or inappropriate care. Second, RAM systematically integrates current scientific evidence with clinical appraisal. By combining a structured literature review with panel-based assessments, RAM ensures that the resulting QIs are both evidence-informed and context-sensitive. Third, the RAM process provides a well-defined rating and consensus structure, including standardised scoring and clear thresholds for agreement and disagreement. While RAM is not a Delphi method in the strict sense, it incorporates key Delphi features such as multiple rating rounds or anonymised feedback between rounds [12, 35].

Following the evidence synthesis conducted in the preceding systematic review [3], the present paper reports the results of a three-round RAND-modified Delphi consensus process that aimed to finalise the development of a routine-data-compatible QI set for the ambulatory care provided to patients with KOA and HOA before TJA in Germany. To achieve this, this Delphi process was conducted between 17 October 2022 and 19 December 2022, consisting of an initial online questionnaire (Round 1), a virtual panel meeting with an additional online questionnaire (Round 2), and a final online questionnaire (Round 3).

### Recruitment of the Delphi panel

The recruitment strategy aimed to assemble a multidisciplinary Delphi panel of seven to 15 members to ensure sufficient diversity while maintaining a structured setting that would allow all panellists to participate actively [12]. Ensuring multidisciplinary representation was intended to facilitate a comprehensive evaluation of the QIs identified in the systematic review. The recruitment strategy aimed to assemble a panel comprising health care professionals – including physicians from general medicine, orthopaedics, physical and rehabilitative medicine, radiology, and physical therapists – as well as patient advocacy organisation representatives. Recruitment was conducted across Germany to ensure a balanced panel in terms of geographic distribution, including health care professionals from both rural and urban areas, as well as gender representation. We aimed to include health care professionals from both inpatient and outpatient settings. To ensure relevant expertise, health care professionals were required to have a minimum of 15 years of experience in their respective fields, while patient advocacy organisation representatives were selected based on their active involvement within patient organisations.

The study team distributed recruitment emails using their professional networks. Due to the diversity of contacts, this included individual physicians and medical societies to reach a broad and relevant audience. Financial incentives were offered to participants for their involvement in the virtual panel meeting (Round 2). In total, 239 emails were distributed until the final panel composition was achieved.

### Selection of quality indicators

The objective of the preceding systematic review was to comprehensively identify all internationally available QIs and guideline recommendations relevant to the ambulatory care of patients with KOA or HOA before TJA, with the aim of extracting QIs that are both applicable to the German health care context and quantifiable using routine data from German SHIs. This review resulted in 20 QI sets and 35 guidelines, from which 24 routine data-based process QIs were synthesised [3].

To support a pragmatic consensus approach, two physicians (one general practitioner and one orthopaedic specialist), neither of whom participated in the subsequent Delphi process, conducted a pre-selection of these systematic-review-derived QIs. Drawing on their clinical expertise in KOA and HOA care in Germany and scientific expertise in the development of quality assurance measures, the two physicians reviewed and discussed each QI. Only those QIs with clear potential to transparently measure the quality of care, and thus to provide informative feedback for health care providers, were included in the first Delphi round.

### Online questionnaire

We administered an electronic questionnaire for each of the three Delphi rounds using the online software LimeSurvey (Version 5.6.25). The introduction to each questionnaire informed panellists about the aim and structure of the Delphi round, the QI rating criteria, and guidelines on anonymity and confidentiality [6, 15].

**Fig. 1:**
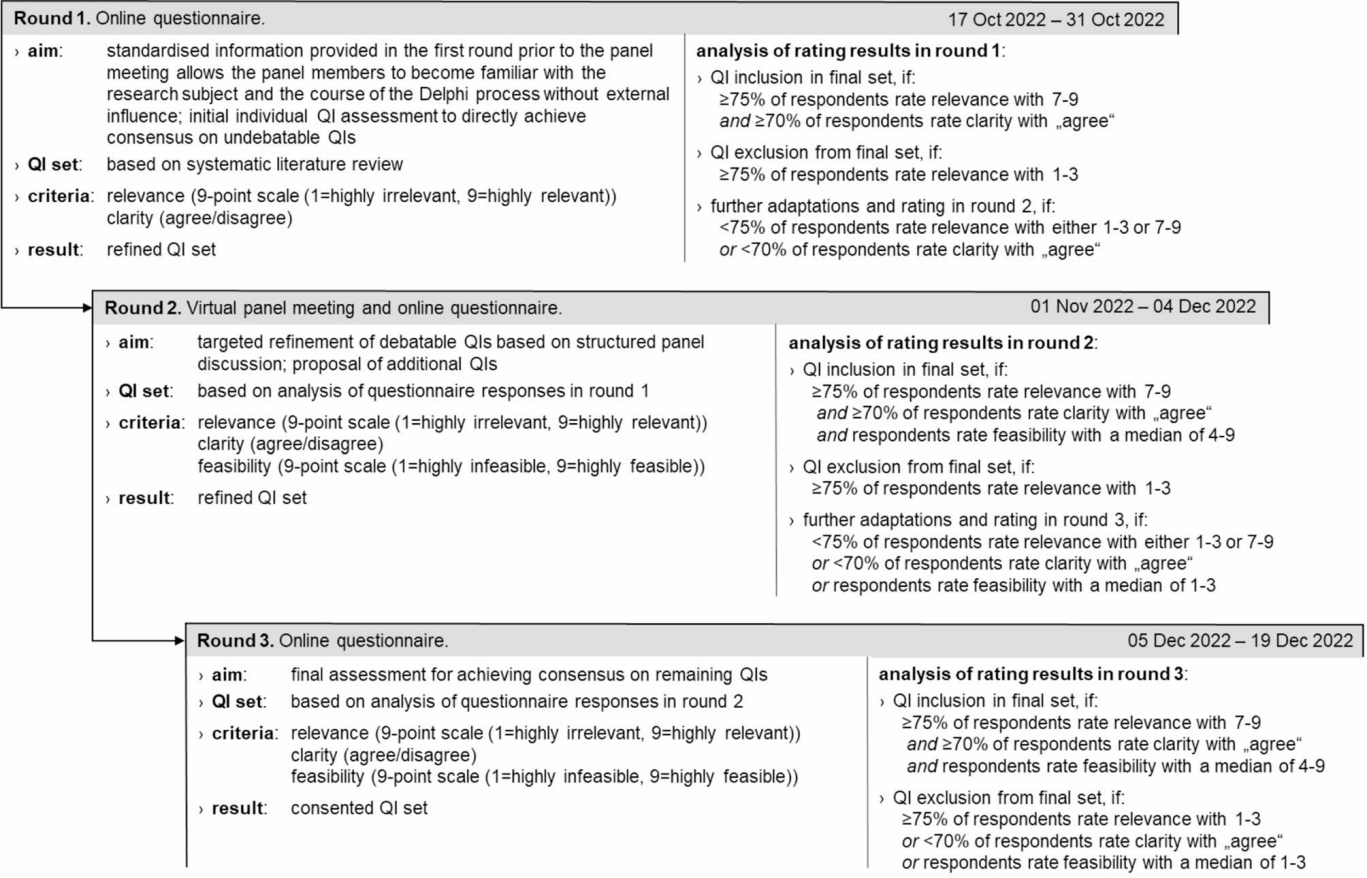
Flow chart of the three-round Delphi process

Following the methodological recommendations of the Institute for Applied Quality Improvement and Research in Health Care GmbH (aQua Institute), we developed a specification sheet for each QI that provided details on the underlying evidence and routine-data-compatible quantification. Panellists used these sheets as a reference when rating the QIs. Throughout the three Delphi rounds, panellists rated the QIs based on the criteria (i) relevance, (ii) clarity and (iii) feasibility. We applied criterion-specific consensus thresholds aligned with established frameworks for medical guideline and QI development in Germany (see Fig. 1) [19, 40]:

i. Relevance: During Rounds 1–3, panellists assessed whether a QI could provide meaningful insights into ambulatory KOA and HOA care in Germany. Relevance was considered higher if a QI could differentiate between the performance of different health care providers, offer substantial benefits compared to potential risks, and be directly influenced by health care providers. Relevance was rated as a global measure on a 9-point scale (1 = highly irrelevant, 9 = highly relevant). A QI was considered relevant if at least 75% of panel ratings fell within the range of 7 to 9, consistent with the aQua Institute’s methodology for QI development [19].
ii. Clarity: During Rounds 1–3, panellists also assessed whether the operationalisation of a QI would be clear and unambiguous, minimising room for interpretation. Clarity was rated as a global measure on a dichotomous scale (agree/disagree). A QI was deemed clear if at least 70% of participants agreed. This threshold for clarity reflects the Association of the Scientific Medical Societies in Germany (AWMF) guideline’s classification of consensus strength, where majority agreement is defined as agreement by more than 50% up to 75% of panellists [40]. Based on this, the study team predefined a minimum threshold of 70% agreement to fulfill the clarity criterion, indicating a strong level of agreement among panellists.
iii. Feasibility: During Rounds 2–3, panellists assessed whether a QI was feasible for routine-data-compatible quantification. In doing so, they were instructed to consider whether potential implementation barriers were appropriately addressed and whether documentation errors could be mitigated. Feasibility was rated as a global measure on a 9-point scale (1 = highly infeasible, 9 = highly feasible). A median score of ≥ 4 indicates a QI to be feasible, following the aQua Institute’s methodology [19].

In addition to scale ratings, the questionnaire included a section for panellists to propose modifications to existing QIs or new, additional QIs. If a proposal received unanimous support, the change was implemented accordingly. In addition to the questionnaire, panellists received a supplementary document with anonymised feedback on the previous round’s panel ratings, presented as criterion-specific bar charts. This allowed the panellists to compare their individual QI ratings to those of the entire panel. The supplementary document also included example quantifications for each QI to demonstrate its practical application and thus support the rating process.

To ensure clarity and consistency [16], a pre-test of the first-round questionnaire was conducted by an orthopaedic surgeon specialised in joint endoprosthetics, who participated in the Delphi process, and a health scientist, who did not. Both had experience in the development of evidence-based guideline recommendations or quality assurance measures.

In all three rounds, panellists received a personalised direct link to the questionnaire via email and were given two weeks to complete it. Email reminders were sent at regular intervals until the submission deadline. Non-responders from previous rounds were not excluded from subsequent rounds and continued to receive the supplementary document containing results from each completed round. Throughout the Delphi process, panellists had the opportunity to contact the research team for clarifications, ensuring a shared understanding of the aims and procedures of the study.

### Delphi process

The first Delphi round aimed to provide panellists with detailed information about the research objectives, the purpose of the Delphi process, and the methodological approach. Panellists rated the QIs derived from the preceding systematic review for their relevance and clarity. QIs that did not meet the predefined consensus thresholds were carried forward to the second round. Suggestions for QI modifications were compiled, refined and incorporated into the next round.

The second Delphi round aimed to facilitate consensus by refining the remaining QIs based on panel input. To achieve this, panellists participated in a virtual meeting using the video communication platform Zoom (Version 5.11.11) [12]. At the beginning of the meeting, a researcher introduced a code of practice to promote equal participation among all panellists and to minimise the risk of dominant individuals disproportionately influencing the discussion [6, 15]. This code of practice included the following principles: (1) in response to closed questions (e.g., suggestions for new QIs), silence was interpreted as agreement unless explicitly stated otherwise; (2) participants were encouraged to express disagreement or alternative views at any time; and (3) if the discussion became unstructured or dominated by a few individuals, the researcher facilitating the meeting would intervene to manage the speaking order and ensure balanced contributions.

Panellists were encouraged to propose additional QIs. The suggestions and modifications that arose from the discussion were integrated into the second-round questionnaire, which was distributed to the panellists immediately after the meeting. In the questionnaire, panellists anonymously reassessed QIs for relevance, clarity and feasibility. QIs that still lacked consensus were carried forward to the third round, along with further proposed modifications or additions, which we systematically sorted, condensed and incorporated.

The third Delphi round aimed to achieve a final consensus on the routine-data-compatible QI set for pre-TJA ambulatory care of patients with KOA or HOA in Germany. In this round, panellists rated QIs again based on relevance, clarity and feasibility.

### Example QI quantification

In addition to the Delphi process, we performed an exemplary quantification of selected QIs to illustrate their potential applicability in practice. This illustrative step was not part of an implementation study but was intended solely to enhance understanding of the QIs’ feasibility and potential for operationalisation.

QIs were quantified for office-based practices in Bavaria, Germany, based on routinely collected claims data from the statutory health insurer AOK Bavaria. The pseudonymised data included information on patient demographics, diagnoses, outpatient and inpatient services, procedures as well as prescriptions of pharmaceuticals, remedies and medical aids. The observation period lasted from 1 January 2019 to 31 December 2019. To focus on ambulatory care before TJA, only patients ≥ 18 years of age with initial HOA and/or KOA diagnosis (ICD-10-GM: M16 and/or M17, diagnostic certainty: G) were included. A pre-observation period (1 January 2014 to 31 December 2018) was considered for detecting the first KOA or HOA diagnosis. We applied the M2Q criterion to verify diagnosis consistency, requiring that a diagnosis be recorded in at least two different quarters within the observation period. Patients are required to be continuously insured with AOK Bavaria (at least 350 days within the observation period). QI quantification included only office-based practices with physicians from medical specialties relevant to the care of KOA and HOA (general medicine, internal medicine and rheumatology, orthopaedics, orthopaedic surgery, physical and rehabilitative medicine, radiology) that had billing contacts for patients with KOA and HOA during the observation period, using the patient as the unit of analysis. Example QI quantification was conducted using R (Version 4.4.2).

## Results

### Composition of the panel

A total of eight physicians (two general practitioners, two specialists in orthopaedic surgery, one specialist in sports orthopaedics, two specialists in physical and rehabilitative medicine, one radiologist, one physical therapist and one patient advocacy organisation representative from the German Rheumatism League (Deutsche Rheuma-Liga Bundesverband e. V.), a national patient advocacy organisation, accepted the invitation to participate in the Delphi process. Nine panellists completed questionnaires in the first two rounds, and eight panellists did so in the third round (see Table 1).

### Pre-selection of systematic-review-derived QIs

**Fig. 2:**
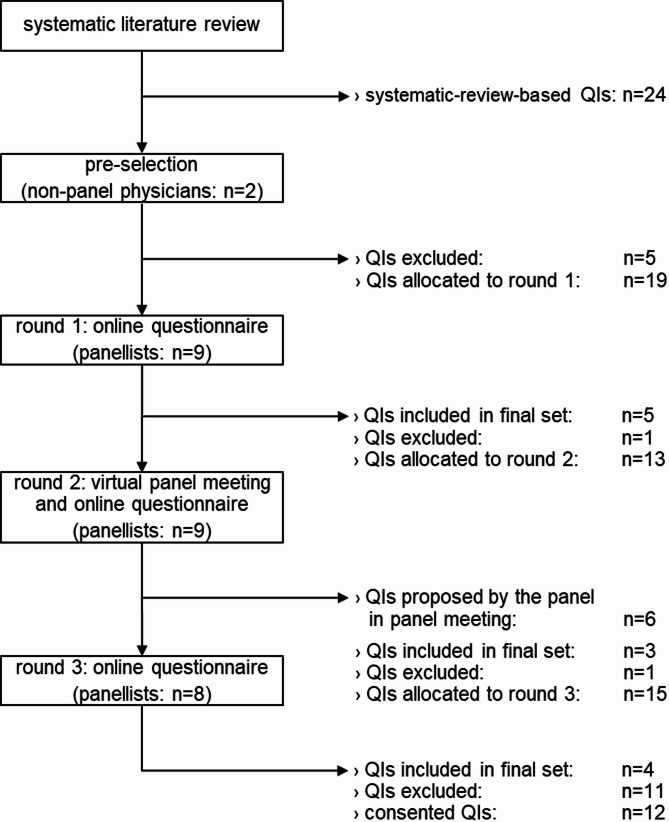
Flow chart of the QI selection process in the three-round Delphi process

During the pre-selection process, the categorisation of QIs from the systematic review into intervention categories was expanded to include a new category: interprofessional collaboration of physicians. One systematic-review-derived QI was subsequently re-categorised into this category (see Table 2). A total of 19 out of the 24 systematic-review-derived QIs were included in the first Delphi round (see Fig. 2). The primary reason for excluding five QIs was their lack of feasibility for routine-data-compatible quantification. For example, the inability to capture the purchase of non-prescription medications in routine data led to the exclusion of QIs related to the prescription of paracetamol, as well as topical and oral non-steroidal anti-inflammatory drugs (NSAIDs). Due to broad similarities in ambulatory treatments, QIs supported by evidence for either KOA or HOA were extrapolated to both conditions.

**Table 1:** Characteristics of the Delphi panel

Panellist identifier	Gender	Professional background	Institutional setting	German federal state of practice	Practice Setting (Urban/Rural)	Participation in Delphi Rounds			
						Round 1	Round 2	Round 3	
P01	Male	General medicine	Office-based outpatient practice	Hesse	Urban	✓	✓	✓	
P02	Male	General medicine	Office-based outpatient practice	Bavaria	Urban	✓	✓	✓	
P03	Male	Orthopaedic surgery	University hospital	Saxony	Urban	✓	✓	✓	
P04	Male	Orthopaedic surgery	Specialist clinic for orthopaedics and rheumatology	Bavaria	Rural	✓	✓		
P05	Female	Sports orthopaedics	University hospital	Baden-Wuerttemberg	Urban	✓	✓	✓	
P06	Female	Physical and rehabilitative medicine	Office-based outpatient practice	Saxony	Urban		✓	✓	
P07	Male	Physical and rehabilitative medicine	Specialist clinic for geriatrics	Bavaria	Urban	✓	✓	✓	
P08	Male	Radiology	Office-based outpatient practice	Bavaria	Urban	✓			
P09	Female	Physical therapy	Physical therapy practice	Baden-Wuerttemberg	Urban	✓	✓	✓	
P10	Female	Patient advocacy	Patient advocacy organisation^1^	-	-	✓	✓	✓	

Legends:

^1^ German Rheumatism League (Deutsche Rheuma-Liga Bundesverband e. V.)

**Table 2:** Results of panellists’ QI ratings in three-round RAND-modified Delphi consensus process

Intervention category	Quality indicator	Derived from the systematic review (*R*) or Delphi panel (D)	Round 1: Online questionnaire.			Round 2: Virtual panel meeting and online questionnaire.				Round 3: Online questionnaire.			Inclusion in final QI set (✓)	
			Relevance^1^		Clarity^2^	Relevance^1^		Clarity^2^	Feasibility^3^	Relevance^1^	Clarity^2^	Feasibility^3^		
			% 7–9	% 1–3	(% agreement)	% 7–9	% 1–3	(% agreement)	(median score)	% 7–9	(% agreement)	(median score)		
Musculo- skeletal appointment	1. If a patient has a diagnosis of KOA/HOA for > 12 months, then there should be documentation of at least one appointment with a qualified physician annually.	R	56	33	100	44	44	100	6	50	100	8		
Diagnostic imaging	2. If a patient is initially diagnosed with KOA/HOA, there must be documentation that the initial KOA/HOA diagnosis was based on radiologic imaging.	R	67	0	100	89	0	100	8				✓	
	3. If the initial KOA/HOA diagnosis has been radiologically examined, then conventional radiography should be used prior to other imaging modalities (MRI, CT, sonography).	R	67	0	89	100	0	100	7,5				✓	
	4. If a KOA/HOA patient is newly prescribed a prescription medication and has no previous radiograph, then a radiograph of the affected joint should be performed.	R	33	56	89	11	78	89	3					
	5. If a KOA/HOA patient has undergone TJA, there should be documentation of radiographic imaging of the affected joint before TJA.	R	56	33	89	56	44	100	7	50	100	5		
	6. Radiological imaging (radiography, MRI, CT, sonography) in KOA/HOA patients should be performed only as medically necessary.	D				56	11	89	6	88	100	6	✓	
Exercise therapy	7. If a patient has a diagnosis of KOA/HOA for > 12 months, then there should be documentation of at least one referral to a physical therapist.	R	78	0	67	78	0	89	5,5				✓	
	8. If a patient has a diagnosis of KOA/HOA for > 3 months, then there should be documentation of at least one referral to a physical therapist within 3 months after the initial KOA/HOA diagnosis.	R	78	0	78								✓	
Medication	9. If a KOA/HOA patient is newly prescribed oral NSAIDs, then ibuprofen should be the first prescribed oral NSAID unless contraindicated.	R	44	11	100	25	38	100	5	14	100	4		
	10. If a KOA/HOA patient has an additional diagnosis of CV risk factors (diabetes mellitus, hypertension, hyperlipidaemia) and is prescribed oral NSAIDs, then naproxen should be the first prescribed oral NSAID unless contraindicated.	R	50	33	78	29	29	100	5	33	100	4,5		
	11. If a KOA/HOA patient has a risk factor for GI bleeding (aged ≥ 75 years, peptic ulcer disease, history of GI bleeding, warfarin use, chronic glucocorticoid use) and is prescribed (COX) non-selective NSAIDs, then misoprostol or a PPI should be prescribed concomitantly.	R	43	0	67	13	25	100	5,5	14	100	6		
	12. If a KOA/HOA patient is prescribed duloxetine, there should be documentation that paracetamol and/or oral NSAIDs and/or weak opioids^4^ have been prescribed before the prescription of duloxetine unless contraindicated.	R	17	83	67									
	13. If a KOA/HOA patient is treated with IA corticosteroids, there should be documentation that paracetamol and/or oral NSAIDs and/or weak opioids^4^ have been prescribed before the application of IA corticosteroids unless contraindicated.	R	50	38	78	56	13	100	5	50	100	5		
	14. If a KOA/HOA patient is treated with IA corticosteroids more than once, then the injection interval should not be shorter than 4 months.	R	63	0	100	57	0	100	5	86	100	5	✓	
	15. If a KOA/HOA patient is unresponsive/ contraindicated to paracetamol and/or oral NSAIDs, then weak opioids^4^ should be prescribed.	R	57	29	67	50	13	100	6	43	100	5		
	16. If a KOA/HOA patient is contraindicated to TJA and has documentation of being prescribed weak opioids^4^, then strong opioids should be prescribed unless contraindicated.	R	56	33	67	67	0	100	6	75	100	7	✓	
TJA preconditions	17. If a KOA/HOA patient has undergone TJA, there should be documentation of a combination of non-pharmacological and pharmacological treatment modalities before TJA.	R	78	11	89								✓	
	18. If a KOA/HOA patient has undergone TJA, there should be documentation of a combination of non-pharmacological and pharmacological treatment modalities for at least 3 months before TJA.	R	100	0	89								✓	
	19. If a KOA/HOA patient is treated with IA corticosteroids, then TJA should be realised 3 months after the last IA injection date.	R	78	0	89								✓	
Inter-professional collaboration	20. If a KOA/HOA patient has an appointment with a physician providing outpatient specialist care, there should be documentation of a preceding referral from a general practitioner.	D				56	11	89	7,5	75	100	7	✓	
	21. If a KOA/HOA patient is registered in a physician network, treatment should be predominantly provided by physicians within that network.	D				33	22	89	6,5	43	100	7		
	22. The number of KOA/HOA patients shared between two physicians should be maximised.	D				56	22	78	6,5	57	100	7		
	23. The treatment of KOA/HOA patients should involve an appropriate number of orthopaedic specialists and orthopaedic surgeons based on patient needs.	D				56	33	78	7,5	63	100	7		
	24. If a KOA/HOA patient is referred to an orthopaedic surgeon, then the waiting time from first referral to appointment should not exceed 3 months.	R	78	11	89								✓	
	25. If a KOA/HOA patient has undergone TJA, there should be documentation of a second opinion procedure before TJA.	D				67	11	100	5	63	88	5		

Legends: COX = cyclooxygenase; CT = computed tomography; CV = cardiovascular; GI = gastrointestinal; HOA = osteoarthritis of the hip; IA = intra-articular; KOA = osteoarthritis of the knee; MRI = magnetic resonance imaging; NSAID = non-steroidal anti-inflammatory drug; PPI = proton pump inhibitor; QI = quality indicator; TJA = total joint arthroplasty

^1^ Relevance was rated on a 9-point scale (1 = highly irrelevant, 9 = highly relevant)

^2^ Clarity was rated on a dichotomous agreement scale (agree/disagree)

^3^ Feasibility was rated on a 9-point scale (1 = highly infeasible, 9 = highly feasible)

^4^ The distinction between weak and strong opioids was made according to the three-level scale of the World Health Organization (WHO) for the management of pain [7]

### Delphi round 1

In the first round, the rating results led to five systematic-review-derived QIs meeting the consensus thresholds for relevance and clarity and were therefore included in the final set for Round 1. One QI was excluded due to a low overall relevance rating. To address the limitations of routine-data-compatible quantifications, panellists suggested adding interpretation notes to the specification sheet of each QI to support a more accurate assessment of a QI’s informative value. The remaining 13 systematic-review-derived QIs without consensus were carried forward to the panel meeting in the second Delphi round.

### Delphi round 2

During a three-hour panel meeting, panellists proposed one additional QI related to diagnostic imaging and five additional QIs related to interprofessional collaboration among physicians in ambulatory KOA and HOA care. We incorporated these QIs into the second-round questionnaire. The proposed QIs aimed to capture the overutilisation of diagnostic imaging procedures, the extent to which general practitioners realised a gatekeeping role, the involvement of an appropriate number of relevant physicians in the care of individual patients, and the use of second-opinion procedures before TJA to assess surgical necessity.

The rating of 19 QIs in the second-round questionnaire led to the inclusion of three systematic-review-derived QIs in the final set for Round 2 and the exclusion of one systematic-review-derived QI. The six QIs proposed by the panel did not reach consensus, and 15 QIs were carried forward into the third Delphi round.

### Delphi round 3

Following the final rating round in the third questionnaire, two refined systematic-review-derived QIs and two panel-proposed QIs were included in the final set. The remaining eleven QIs did not meet criterion-specific consensus thresholds and were excluded.

### Final QI set

The final, consensus-based QI set consisted of twelve routine-data-compatible process QIs related to diagnostic imaging, exercise therapy, medication, TJA preconditions, and interprofessional collaboration in ambulatory KOA and HOA care before TJA (see Table 3).

For diagnostic imaging, the QIs specify that the initial diagnosis of KOA or HOA should be based on radiographic imaging (Q1) and that conventional radiography should be performed before other imaging modalities such as MRI, CT, or sonography (Q2). Additionally, radiological imaging should be conducted only as medically necessary, limiting unnecessary examinations (Q3). In the area of exercise therapy, the QIs require that patients with KOA or HOA for more than twelve months should have at least one documented referral to supervised exercise therapy within the first year following diagnosis (QI4). Similarly, patients with a diagnosis for more than three months should have a documented referral within three months of diagnosis (QI5). For medication, the QIs state that intra-articular (IA) corticosteroid injections should not be administered more frequently than once every six months (QI6). In cases where strong opioids are prescribed due to contraindications to TJA, the use of weak opioids should have been attempted first, unless contraindicated (QI7). Regarding TJA preconditions, the QIs require that patients who have undergone TJA should have documentation of both non-pharmacological and pharmacological treatment modalities before surgery (QI8, QI9). Additionally, TJA should not be performed sooner than three months after an IA corticosteroid injection (QI10). For interprofessional collaboration, QIs state that referrals should be documented before a patient sees an outpatient specialist or orthopaedic surgeon (QI11, QI12).

QIs were mainly excluded because they could not be feasibly quantified using routine data. To improve interpretability, the QI specification sheets were expanded with explanatory notes, as recommended by the panel. These notes primarily help ensure the accurate interpretation of routine-data-based quality information. Further details on the operationalisation of the finalised, consensus-based set of QIs are provided in Appendix 1.

### Example QI quantification

**Fig. 3:**
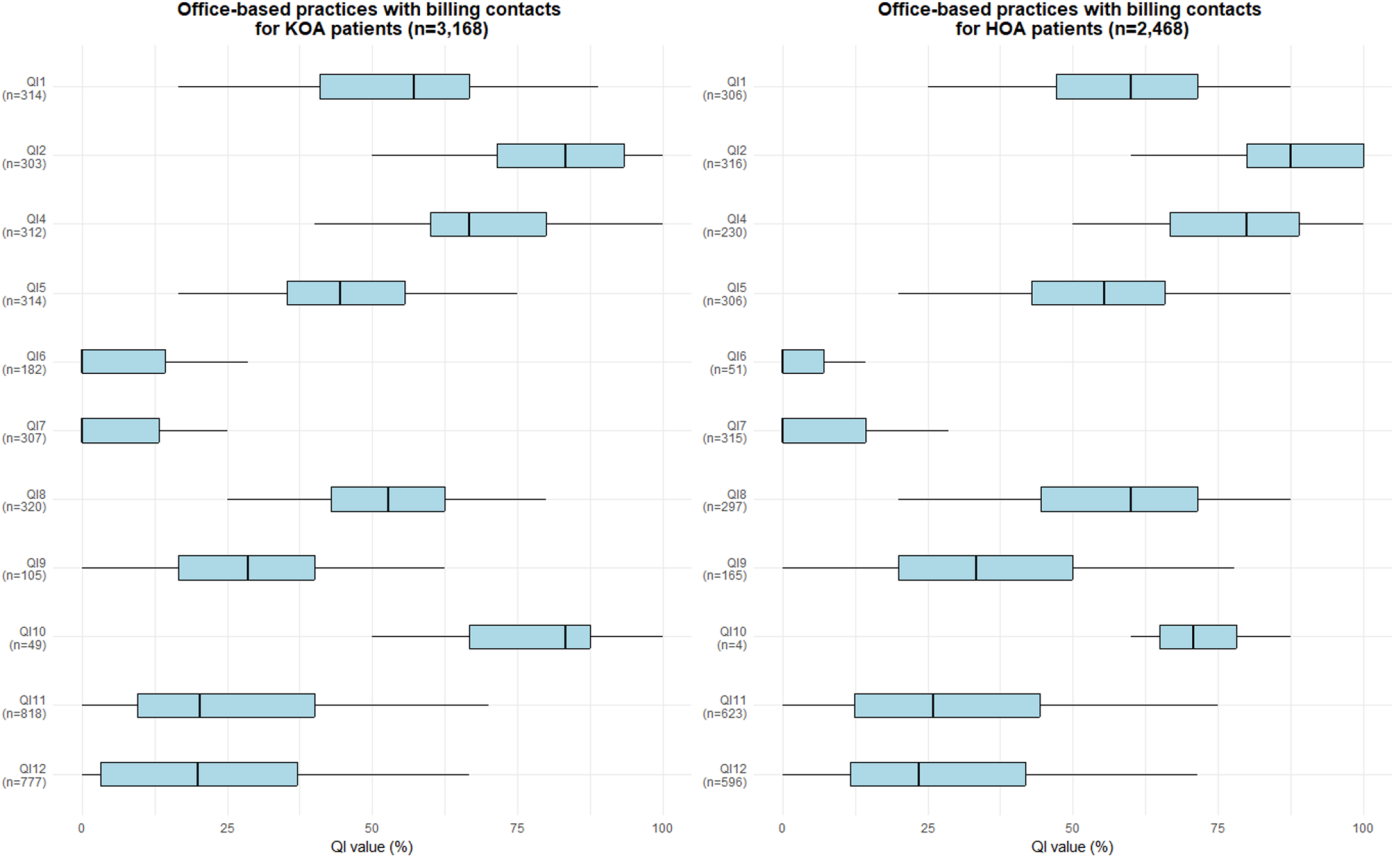
QI values of office-based practices with billing contacts for KOA and HOA patients in Bavaria, Germany. Legends: HOA = osteoarthritis of the hip; KOA = osteoarthritis of the knee; QI = quality indicator. QI identifiers: QI1: initial diagnosis based on radiographic imaging, QI2: radiography as primary imaging modality, QI4: documentation of at least one referral to supervised exercise therapy within 12 months after initial diagnosis, QI5: initiation of supervised exercise therapy within 3 months after initial diagnosis, QI6. minimum duration of 6 months between IA corticosteroid injections, QI7: prescription of strong opioids only after weak opioids have proven ineffective, QI8: TJA after combined non-pharmacological and pharmacological treatment, QI9: minimum duration of 3 months of combined non-pharmacological and pharmacological treatment prior to TJA, QI10: minimum duration of 3 months between the last IA corticosteroid injection and TJA, QI11: specialist physician visit only with referral, QI12: orthopaedic surgeon visit only with referral. Note: QI quantification included only office-based practices with physicians from medical specialties relevant to the care of KOA and HOA (general medicine, internal medicine and rheumatology, orthopaedics, orthopaedics and trauma surgery, physical and rehabilitative medicine, radiology) that had billing contacts for patients with KOA and HOA during the observation period (1 January 2019–31 December 2019), using the patient as the unit of analysis. Only practices with > 4 patients in the QI-specific denominator population in the observation period were considered for the quantification of QI values

Figure 3 presents an example quantification of eleven of the final QIs expressed as proportions. A total of 3,550 practices were included in the analysis. In total, these practices had billing contacts for 261,077 patients, including those diagnosed with KOA (*n* = 195,879), HOA (*n* = 110,587), or both conditions (*n* = 45,389) during the observation period. Only practices with > 4 patients in the QI-specific denominator population in the observation period were considered for the quantification of QI values.

Overall, the boxplot visualisation of QI values reveals considerable variation between practices in adherence to QI recommendations. In KOA care, the median QI value exceeded 50% for five QIs, while in HOA care, this threshold was surpassed for six QIs. Notably, the low values for QI6 (minimum duration of six months between IA corticosteroid injections) and QI7 (prescription of strong opioids only after weak opioids have proven ineffective) stand out. When interpreting QI values, it is important to consider that some QIs were quantifiable for only a small number of practices: In KOA care, one QI was quantifiable in fewer than 100 practices; in HOA care, two QIs met this criterion, with one (QI10: minimum duration of three months between the last IA corticosteroid injection and TJA) being quantifiable in only four practices.

## Discussion

### Development approach

By combining a systematic review with a subsequent Delphi process, RAM provides a reproducible development framework, ensuring that all panellists access a uniform, scientifically substantiated body of evidence, thereby helping prevent disagreements. The participation of ten panellists met the methodological requirements for an effective exchange of perspectives. By including relevant health care professionals involved in KOA and HOA care, the QIs could be tailored to the context of ambulatory KOA and HOA care in Germany, supporting content validity [15].

When comparing studies that combine a systematic review with a subsequent RAND-modified Delphi consensus process for developing QIs for OA care, the proportion of systematic-review-derived QIs included in the final QI set (53%, 10/19) was consistent with findings from MacLean, et al. [27] (56%, 13/23). In the study of Kleudgen, et al. [24], on the other hand, only 25% (1/4) of the QIs derived from the systematic review were agreed upon in the subsequent Delphi process.

Our three-round Delphi approach began with an initial online questionnaire in which each panellist independently assessed the QIs without being influenced by the other panel members’ views. This process allowed certain QIs to reach consensus without the need for further discussion. As a result, the subsequent panel meeting in the second round was able to focus solely on the QIs that had not yet achieved consensus, enabling a more comprehensive and structured discussion of these QIs.

The consensus threshold required for QI inclusion varies across Delphi studies. Previous studies developing KOA- or HOA-specific QIs using a RAND-modified Delphi approach have determined inclusion based on the median of panel ratings, supplemented by a measure of dispersion [1, 2, 10, 17, 24, 27, 29, 30, 41, 44]. In studies using percentage-based agreement thresholds, the required thresholds range from 60 to 80% [20, 26, 28, 42, 45]. Had we required unanimous agreement on the highest possible rating for relevance and clarity across the three Delphi rounds, only one QI would have met the threshold. Using an 80% or 60% agreement threshold would have resulted in the inclusion of five QIs or 14 QIs, respectively, after the third Delphi round. If a simple majority had been deemed sufficient, 19 QIs would have been included. Ultimately, applying consensus thresholds of ≥ 75% for relevance and ≥ 70% for clarity ensured that only robust QIs were included, whereas lower thresholds would have resulted in a larger set with more debatable QIs.

Ten systematic-review-derived QIs reached consensus during the first two Delphi rounds. In contrast, the relatively high number of unresolved QIs included in the final questionnaire reflects the challenge of translating evidence into practical quality measures, particularly when they must be quantifiable using routine data. To address this, the panellists in our Delphi process introduced QI-specific interpretation notes to account for the limitations of the routine data. These notes are intended to help heath care providers interpret each QI and take appropriate action to improve outpatient KOA and HOA care.

### Final QI set

The final QI set can provide information on the type, order and frequency of diagnostic imaging procedures, referrals to supervised exercise therapy, pharmaceutical prescriptions and referrals to specialist physicians, thus depicting essential elements that should be considered prior to TJA. A study that also examined the use of routine data to measure the quality of OA care concluded that despite the increasing availability of routine data, only selected aspects of quality of care can be measured [48]. Crucial factors for patient-specific disease management, such as individual patient preferences, OA severity or individual levels of pain and suffering, are not captured by routine data. Therefore, further measurement of quality of care still requires the collection of clinical data. Although the QIs developed here do not represent a complete assessment of ambulatory KOA and HOA care prior to TJA, the vast majority of these QIs can be generated for a large number of patients and can therefore be used for targeted improvement and serial measurement of care to monitor the impact of interventions.

Although the adaptations made to the systematic-review-derived QIs in our Delphi process were minor, they highlight the importance of tailoring evidence to the specific care context. Most importantly, panellists repeatedly emphasised the need to prioritise individual patient needs and preferences throughout the often long-term disease course, particularly when considering TJA.

QI-based feedback provides an evidence-based framework for identifying deficits in health care provision and, in doing so, can serve as the basis for discussing options for improvement. Sharing QI results among health care providers from different disciplines may facilitate the definition of process standards in the ambulatory care provided to patients with KOA and HOA before TJA in Germany [13, 21].

Although the example QI quantifications reflect standard ambulatory care in Bavaria, some QIs could only be calculated for a few office-based practices. Therefore, when interpreting QI results, it is essential to consider the underlying numerator and denominator populations. The low values for QI6 and QI7 could indicate that care delivery was not compliant with clinical standards. However, low values in QI6 may also cast doubt on the feasibility of routine-data-compatible operationalisation, as the relevant billing code for IA corticosteroid application may also cover injections with other substances or joint punctures.

### Limitations

Our study has several important limitations. First, it is important to note that the outcome of a consensus process cannot be equated with generalisability [36]. Because the Delphi method does not rely on a random sample representative of the target population, the credibility of its findings depends largely on the composition of the panel [5, 46]. Selection bias may have occurred, as participation was probably limited to those with a particular motivation to participate and engage in the process. Additionally, panellists’ level of engagement and commitment may have increased if they perceived the consensus outcome as directly impacting their own practice [23]. Moreover, to account for panel-specific influences, potential biases arising from group dynamics in the panel meeting (e.g., authority bias, in-group bias) should be considered. Finally, providing rating results alongside the questionnaire may have encouraged conformity bias. However, it can be argued that a tendency towards agreement is an inherent feature of the Delphi process, which is designed to produce consensus-based opinion [39].

## Conclusion

The Delphi process resulted in a final set of twelve evidence- and consensus-based, routine-data-compatible QIs for ambulatory KOA and HOA care before TJA. The QIs cover key areas such as diagnostic imaging, exercise therapy, pharmacological treatment, prerequisites for TJA, and interprofessional collaboration. They are intended to support health care providers in monitoring and improving care processes. However, to ensure the effective use and interpretation of these QIs, health care providers may require additional training on the use of routine data for quality measurement. Future research should evaluate the implementation of the QI set in routine practice, including its application in audit and feedback interventions to assess acceptance and its potential to identify variation in care quality.

**Table 3:** Final, consensus-based routine-data-compatible QI set for ambulatory care of KOA and HOA before TJA

Intervention category	Quality indicator^1^
Diagnostic imaging	1. If a patient is newly diagnosed with KOA/HOA, the diagnosis should be based on radiographic imaging.
	2. If the initial diagnosis of KOA/HOA was based on imaging, conventional radiography should have been used before other imaging modalities (MRI, CT, sonography). 3. Imaging in KOA/HOA patients should be performed only as medically necessary (measured as the number of radiographic/MRI/CT/sonographic examinations performed in KOA/HOA patients).
Exercise therapy	4. If a patient has had a diagnosis of KOA/HOA for > 12 months, there should be documentation of at least one referral to supervised exercise therapy within 12 months after the initial KOA/HOA diagnosis.
	5. If a patient has had a diagnosis of KOA/HOA for > 3 months, there should be documentation that the first referral to supervised exercise therapy took place within 3 months after the initial KOA/HOA diagnosis.
Medication	6. If a KOA/HOA patient receives multiple IA corticosteroid injections, the interval between the injections should not be shorter than 6 months.
	7. If a KOA/HOA patient is contraindicated for TJA and has documentation of being prescribed strong opioids^2^, there should be documentation that weak opioids were prescribed first, unless contraindicated.
TJA preconditions	8. If a KOA/HOA patient has undergone TJA, there should be documentation of a combination of non-pharmacological and pharmacological treatment modalities before surgery.
	9. If a KOA/HOA patient has undergone TJA, there should be documentation that a combination of non-pharmacological and pharmacological treatment modalities was provided for at least 3 months before surgery.
	10. If a KOA/HOA patient is treated with IA corticosteroids, TJA should not be performed sooner than 3 months after the last IA corticosteroid injection.
Inter- professional collaboration	11. If a KOA/HOA patient has an appointment with a physician providing outpatient specialist care, there should be documentation of a prior referral.
	12. If a KOA/HOA patient has an appointment with an orthopaedic surgeon, there should be documentation of a prior referral.

Legends: CT = computed tomography; HOA = osteoarthritis of the hip; IA = intra-articular; KOA = osteoarthritis of the knee; MRI = magnetic resonance imaging, QI = quality indicator; TJA = total joint arthroplasty

^1^ See Appendix 1 for detailed information on the operationalisation provided in the QI specification sheets

^2^ The distinction between weak and strong opioids is made according to the three-level scale of the World Health Organization (WHO) for the management of pain [7]

## Electronic supplementary material

Below is the link to the electronic supplementary material.

Supplementary Material 1

## Data Availability

The data that support the findings of this study are available from the AOK Bavaria but restrictions apply to the availability of these data, which were used under license for the current study, and so are not publicly available. Data is however available from the authors upon reasonable request and with permission of the AOK Bavaria and their regulatory authority.

## Abbreviations

aQua: Institute institute for applied quality improvement and research in health
Care: GmbH
AWMF: Association of the scientific medical societies in Germany
CT: Computed tomography
CV: Cardiovascular
GI: Gastrointestinal
HOA: Osteoarthritis of the hip
IA: Intra-articular
KOA: Osteoarthritis of the knee
MRI: Magnetic resonance imaging
NSAID: Non-steroidal anti-inflammatory drug
OA: Osteoarthritis
OECD: Organisation for economic co-operation and development
PPI: Proton pump inhibitors
QI: Quality indicator
RAM: RAND/UCLA appropriateness method
SHI: Statutory health insurer
TJA: Total joint arthroplasty
WHO: World health organization
