# Supplementary material for: Development of routine-data-compatible quality indicators for the management of osteoarthritis of the knee and hip in ambulatory care: a RAND-modified Delphi consensus process
Source: J Orthop Surg Res. 2025 Jul 26;20:711. doi: 10.1186/s13018-025-06127-x (PMC12296632; doi:10.1186/s13018-025-06127-x)
Supplement: Supplementary file 1 — Supplementary Material 1 [file 13018_2025_6127_MOESM1_ESM.docx]

**APPENDIX 1.**

QI specification sheets.

[OSTEOARTHRITIS OF THE HIP 2](#_Toc194571378)

[QI1 Initial diagnosis using radiographic examination 2](#_Toc194571379)

[QI2 Radiography as primary imaging diagnostics 3](#_Toc194571380)

[QI3a Number of radiographic examinations performed per patient 4](#_Toc194571381)

[QI3b Number of MRI examinations performed per patient 5](#_Toc194571382)

[QI3c Number of CT examinations performed per patient 6](#_Toc194571383)

[QI3d Number of sonographic examinations performed per patient 7](#_Toc194571384)

[QI4 Documentation of at least one referral to supervised exercise therapy within 12 months after initial diagnosis 8](#_Toc194571385)

[QI5 Initiation of supervised exercise therapy within 3 months after initial diagnosis 9](#_Toc194571386)

[QI6 Minimum duration of 6 months between IA corticosteroid injections 10](#_Toc194571387)

[QI7 Prescription of strong opioids only after weak opioids have proven ineffective 11](#_Toc194571388)

[QI8 TJA after combined non-pharmacological and pharmacological treatment 13](#_Toc194571389)

[QI9 Minimum duration of 3 months of combined non-pharmacological and pharmacological treatment prior to TJA 14](#_Toc194571390)

[QI10 Minimum duration of 3 months between the last IA corticosteroid injection and TJA 15](#_Toc194571391)

[QI11 Specialist physician visit only with referral 16](#_Toc194571392)

[QI12 Orthopaedic surgeon visit only with referral 17](#_Toc194571393)

[OSTEOARTHRITIS OF THE KNEE 18](#_Toc194571394)

[QI1 Initial diagnosis using radiographic examination 18](#_Toc194571395)

[QI2 Radiography as primary imaging diagnostics 19](#_Toc194571396)

[QI3a Number of radiographic examinations performed per patient 20](#_Toc194571397)

[QI3b Number of MRI examinations performed per patient 21](#_Toc194571398)

[QI3c Number of CT examinations performed per patient 22](#_Toc194571399)

[QI3d Number of sonographic examinations performed per patient 23](#_Toc194571400)

[QI4 Documentation of at least one referral to supervised exercise therapy within 12 months after initial diagnosis 24](#_Toc194571401)

[QI5 Initiation of supervised exercise therapy within 3 months after initial diagnosis 25](#_Toc194571402)

[QI6 Minimum duration of 6 months between IA corticosteroid injections 26](#_Toc194571403)

[QI7 Prescription of strong opioids only after weak opioids have proven ineffective 27](#_Toc194571404)

[QI8 TJA after combined non-pharmacological and pharmacological treatment 29](#_Toc194571405)

[QI9 Minimum duration of 3 months of combined non-pharmacological and pharmacological treatment prior to TJA 30](#_Toc194571406)

[QI10 Minimum duration of 3 months between the last IA corticosteroid injection and TJA 31](#_Toc194571407)

[QI11 Specialist physician visit only with referral 32](#_Toc194571408)

[QI12 Orthopaedic surgeon visit only with referral 33](#_Toc194571409)

[LIST OF ABBREVIATIONS 34](#_Toc194571410)

[REFERENCES 35](#_Toc194571411)

# OSTEOARTHRITIS OF THE HIP

## QI1 Initial diagnosis using radiographic examination

| **QI1** | Initial diagnosis using radiographic examination |
| --- | --- |
| **Description** | If a patient is newly diagnosed with HOA, the diagnosis should be based on radiographic imaging. |
| **Note on interpretation** | Not meeting the quality indicator’s requirements may be medically justified in individual cases. The assignment of the billing code for the radiographic examination to the corresponding HOA diagnosis is possible to a limited extent only. In addition, the functional limitations and clinical complaints of the patient are decisive for a treatment decision. |
| **Numerator** | HOA patients with a billing code for a radiographic examination^1^ in the same, the preceding or the following billing quarter as the initial diagnosis  *^1^EBM: 34234, 34235, 34237 and diagnosis ICD (M16*,* *diagnostic certainty: G) in the same billing case* |
| **Denominator** | HOA patients with initial diagnosis ICD (M16*, diagnostic certainty: G) |
| **Exclusion criteria** | - |
| **Quality objective** | *Aim: high value*  The QI represents the proportion of HOA patients with radiographic examination used for determining the initial diagnosis.  A high proportion of HOA patients meeting the quality indicator’s requirements may indicate an increased treatment safety, as a reliable diagnosis ensures targeted and needs-based coordination of medical care. |
| **Rationale** | In addition to the clinical examination, the radiographic examination is used to classify the progression of the osteoarthritis disease. This can lead to an improved treatment outcome and progress monitoring. |
| **Original wording** | Die Diagnose der Gonarthrose soll klinisch und radiologisch gestellt werden. [AWMF (2018), S. 8] |
| **Indicator variations** | - |
| **Dimension of quality** | Process |
| **Risk adjustment** | No risk adjustment |
| **Data source** | Routine data of statutory health insurances |
| **Literature references** | AWMF (2019), AWMF (2018) |

## QI2 Radiography as primary imaging diagnostics

| **QI2** | Radiography as primary imaging diagnostics |
| --- | --- |
| **Description** | If the initial diagnosis of HOA was based on imaging, conventional radiography should have been used before other imaging modalities (MRI, CT, sonography). |
| **Note on interpretation** | Not meeting the quality indicator’s requirements may be medically justified in individual cases. The assignment of the billing code for the radiographic examination to the corresponding HOA diagnosis is possible to a limited extent only. |
| **Numerator** | HOA patients with a billing code for a radiographic examination^1^ in the same, the preceding or the following billing quarter as the initial diagnosis  *^1^EBM: 34234, 34235, 34237 and diagnosis ICD (M16*,* *diagnostic certainty: G) in the same billing case* |
| **Denominator** | HOA patients with a billing code for a radiologic examination (radiography, MRI, CT, sonography)^2^ in the same, the preceding or the following billing quarter as the initial diagnosis  *^2^EBM: 34237, 34234, 34235, 33050, 34350, 34450 and diagnosis ICD (M16*,* *diagnostic certainty: G) in the same billing case* |
| **Exclusion criteria** | - |
| **Quality objective** | *Aim: high value*  The QI represents the proportion of HOA patients for whom the radiological diagnosis was first made by radiographic examination before other imaging procedures (sonography, CT, MRI) were considered.  A high proportion of HOA patients meeting the quality indicator’s requirements may indicate an increased appropriateness, as other imaging procedures (sonography, CT, MRI) should only be performed in cases of unclear joint symptoms, discrepancy between clinical and radiographic findings or lack of therapeutic success. |
| **Rationale** | Radiographic examination is an inexpensive and easily performable procedure for assessing the severity of osteoarthritis and the associated possible surgical procedures and expected complications. |
| **Original wording** | If imaging is needed, conventional (plain) radiography should be used before other modalities. To make additional diagnoses, soft tissues are best imaged by US or MRI and bone by CT or MRI. [EULAR (2017) S. 1486] |
| **Indicator variations** | - |
| **Dimension of quality** | Process |
| **Risk adjustment** | No risk adjustment |
| **Data source** | Routine data of statutory health insurances |
| **Literature references** | AWMF (2021), SIR (2019), AWMF (2019), AWMF (2018), EULAR (2017), NHG (2008) |

## QI3a Number of radiographic examinations performed per patient

| **QI3a** | Number of radiographic examinations performed per patient |
| --- | --- |
| **Description** | Radiographic examination in HOA patients should be performed only as medically necessary. |
| **Note on interpretation** | Not meeting the quality indicator’s requirements may be medically justified in individual cases. The assignment of the billing code for the radiographic examination to the corresponding HOA diagnosis is possible to a limited extent only. Once there is a reliable diagnosis, treatment decisions should be based primarily on the clinical examination, not the radiological examination. |
| **Numerator** | Number of performed radiographic examinations^1^  *^1^EBM: 34237, 34234, 34235 and diagnosis ICD (M16*,* *diagnostic certainty: G) in the same billing case* |
| **Denominator** | Number of HOA patients with at least one radiographic examination^1^ |
| **Exclusion criteria** | - |
| **Quality objective** | *Aim: low value*  The QI represents the number of radiographic examinations performed per HOA patient (with at least one radiographic examination performed).  A high number of radiographic examinations performed per HOA patient compared to other health service providers may indicate inadequate coordination or overuse. |
| **Rationale** | Radiological imaging in HOA patients should be performed as needed and appropriate. A high rate of radiographic examinations per patient may be an indication of overprovision. |
| **Original wording** | - |
| **Indicator variations** | - |
| **Dimension of quality** | Process |
| **Risk adjustment** | No risk adjustment |
| **Data source** | Routine data of statutory health insurances |
| **Literature references** | (based on Delphi panel consensus) |

## QI3b Number of MRI examinations performed per patient

| **QI3b** | Number of MRI examinations performed per patient |
| --- | --- |
| **Description** | MRI examinations in HOA patients should be performed only as medically necessary. |
| **Note on interpretation** | Not meeting the quality indicator’s requirements may be medically justified in individual cases. The assignment of the billing code for the MRI examination to the corresponding HOA diagnosis is possible to a limited extent only. Once there is a reliable diagnosis, treatment decisions should be based primarily on the clinical examination, not the radiological examination. |
| **Numerator** | Number of performed MRI examinations^1^  *^1^EBM: 34450 and diagnosis ICD (M16*,* *diagnostic certainty: G) in the same billing case* |
| **Denominator** | Number of HOA patients with at least one MRI examination^1^ |
| **Exclusion criteria** | - |
| **Quality objective** | *Aim: low value*  The QI represents the number of MRI examinations performed per HOA patient (with at least one MRI examination performed).  A high number of MRI examinations performed per HOA patient compared to other health service providers may indicate inadequate coordination or overuse. |
| **Rationale** | Radiological imaging in HOA patients should be performed as needed and appropriate. A high rate of MRI examinations per patient may be an indication of overprovision. |
| **Original wording** | - |
| **Indicator variations** | - |
| **Dimension of quality** | Process |
| **Risk adjustment** | No risk adjustment |
| **Data source** | Routine data of statutory health insurances |
| **Literature references** | (based on Delphi panel consensus) |

## QI3c Number of CT examinations performed per patient

| **QI3c** | Number of CT examinations performed per patient |
| --- | --- |
| **Description** | CT examinations in HOA patients should be performed only as medically necessary. |
| **Note on interpretation** | Not meeting the quality indicator’s requirements may be medically justified in individual cases. The assignment of the billing code for the CT examination to the corresponding HOA diagnosis is possible to a limited extent only. Once there is a reliable diagnosis, treatment decisions should be based primarily on the clinical examination, not the radiological examination. |
| **Numerator** | Number of performed CT examinations^1^  *^1^EBM: 34350 and diagnosis ICD (M16*,* *diagnostic certainty: G) in the same billing case* |
| **Denominator** | Number of HOA patients with at least one CT examination^1^ |
| **Exclusion criteria** | - |
| **Quality objective** | *Aim: low value*  The QI represents the number of CT examinations performed per HOA patient (with at least one CT examination performed).  A high number of CT examinations performed per HOA patient compared to other health service providers may indicate inadequate coordination or overuse. |
| **Rationale** | Radiological imaging in HOA patients should be performed as needed and appropriate. A high rate of CT examinations per patient may be an indication of overprovision. |
| **Original wording** | - |
| **Indicator variations** | - |
| **Dimension of quality** | Process |
| **Risk adjustment** | No risk adjustment |
| **Data source** | Routine data of statutory health insurances |
| **Literature references** | (based on Delphi panel consensus) |

## QI3d Number of sonographic examinations performed per patient

| **QI3d** | Number of sonographic examinations performed per patient |
| --- | --- |
| **Description** | Sonographic examinations in HOA patients should be performed only as medically necessary. |
| **Note on interpretation** | Not meeting the quality indicator’s requirements may be medically justified in individual cases. The assignment of the billing code for the sonographic examination to the corresponding HOA diagnosis is possible to a limited extent only. Once there is a reliable diagnosis, treatment decisions should be based primarily on the clinical examination, not the radiological examination. |
| **Numerator** | Number of performed sonographic examinations^1^  *^1^EBM: 33050 and diagnosis ICD (M16*,* *diagnostic certainty: G) in the same billing case* |
| **Denominator** | Number of HOA patients with at least one sonographic examination^1^ |
| **Exclusion criteria** | - |
| **Quality objective** | *Aim: low value*  The QI represents the number of sonographic examinations performed per HOA patient (with at least one sonographic examination performed).  A high number of sonographic examinations performed per HOA patient compared to other health service providers may indicate inadequate coordination or overuse. |
| **Rationale** | Radiological imaging in HOA patients should be performed as needed and appropriate. A high rate of sonographic examinations per patient may be an indication of overprovision. |
| **Original wording** | - |
| **Indicator variations** | - |
| **Dimension of quality** | Process |
| **Risk adjustment** | No risk adjustment |
| **Data source** | Routine data of statutory health insurances |
| **Literature references** | (based on Delphi panel consensus) |

## QI4 Documentation of at least one referral to supervised exercise therapy within 12 months after initial diagnosis

| **QI4** | Documentation of at least one referral to supervised exercise therapy within 12 months after initial diagnosis |
| --- | --- |
| **Description** | If a patient has had a diagnosis of HOA for >12 months, there should be documentation of at least one referral to supervised exercise therapy within 12 months after the initial HOA diagnosis. |
| **Note on interpretation** | Not meeting the quality indicator’s requirements may be medically justified in individual cases. The assignment of the billing code for the radiographic examination to the corresponding HOA diagnosis is possible to a limited extent only. Instructional activities beyond the billing-relevant provision of health services (e.g., instructions for self-management), which also contribute to the quality of care, are not depicted. |
| **Numerator** | HOA patients with initial diagnosis ICD (M16*, diagnostic certainty: G) and at least one prescription for exercise therapy^1^ within 12 months after initial diagnosis  *^1^relevant billing codes for prescription therapies: X03XX – X06XX and X09XX, X10XX except X0607, outpatient rehabilitation: TFA: T07C, course fees: TFA: T047, functional training: EBM: 01621* |
| **Denominator** | HOA patients with initial diagnosis ICD (M16*, diagnostic certainty: G) |
| **Exclusion criteria** | - |
| **Quality objective** | *Aim: high value*  The QI represents the proportion of HOA patients who were referred to supervised exercise therapy at least once within a maximum of 12 months following the initial diagnosis.  A high proportion of HOA patients meeting the quality indicator’s requirements may indicate that supervised exercise therapy as a core element of osteoarthritis treatment was initiated at an early stage of disease. By this, strengthening of the muscles surrounding the joint is initiated and carried out correctly under professional guidance. |
| **Rationale** | Exercise therapy has a positive effect on pain relief, functional improvement and quality of life. HOA patients should therefore start exercise therapy to strengthen the stabilising muscles surrounding the joint as early as possible to help slow down the progression of osteoarthritis. Targeted, individualised exercise therapy is one of the core elements of osteoarthritis therapy and should be professionally guided. |
| **Original wording** | IF an ambulatory NH Resident has had a diagnosis of symptomatic osteoarthritis of the knee for >12 months and has no contraindication to exercise and is physically and mentally able to exercise, THEN there should be evidence that a directed or supervised strengthening or aerobic exercise program was prescribed at least once since the time of diagnosis. [Zingmond DS, et al. (2009), supplementary material, S. 46] |
| **Indicator variations** | IF an ambulatory NH resident has a diagnosis of symptomatic knee osteoarthritis for >3 months, has no contraindication to exercise, and is physically and mentally able to exercise THEN there should be evidence that a directed or supervised strengthening or aerobic exercise program was prescribed at least once since the time of diagnosis. [Saliba D (2004), S. 304]  Providers caring for patients with symptoms of hip or knee OA should recommend exercise programs at least once in 2 years. [McGlynn EA, et al. (2003), Appendix A, S. 39; Asch SM (2004), S. W-200]  Providers caring for patients with symptoms of hip or knee OA should recommend both of the following at least once in 2 years:  a. exercise programs for persons with hip or knee OA;  b. weight loss among persons with knee OA and a BMI >25. [Moore A (2000), S. 260] |
| **Dimension of quality** | Process |
| **Risk adjustment** | No risk adjustment |
| **Data source** | Routine data of statutory health insurances |
| **Literature references** | Doubova SV, et al. (2015), Zingmond DS, et al. (2009), Smith KL, et al. (2007) |

## QI5 Initiation of supervised exercise therapy within 3 months after initial diagnosis

| **QI5** | Initiation of supervised exercise therapy within 3 months after initial diagnosis |
| --- | --- |
| **Description** | If a patient has had a diagnosis of HOA for >3 months, there should be documentation that the first referral to supervised exercise therapy took place within 3 months after the initial HOA diagnosis. |
| **Note on interpretation** | Not meeting the quality indicator’s requirements may be medically justified in individual cases. The assignment of the billing code for the radiographic examination to the corresponding HOA diagnosis is possible to a limited extent only. Advising and informing activities beyond the billing-relevant provision of health services (e.g. instructions for self-management), which also contribute to the quality of care, are not depicted. |
| **Numerator** | HOA patients with initial diagnosis ICD (M16*, diagnostic certainty: G) and at least one prescription for exercise therapy^1^ within 3 months after initial diagnosis  *^1^relevant billing codes for prescription therapies: X03XX – X06XX and X09XX, X10XX except X0607, outpatient rehabilitation: TFA: T07C, course fees: TFA: T047, functional training: EBM: 01621* |
| **Denominator** | HOA patients with initial diagnosis ICD (M16*, diagnostic certainty: G) |
| **Exclusion criteria** | - |
| **Quality objective** | *Aim: high value*  The QI represents the proportion of HOA patients who were referred to supervised exercise therapy at least once within a maximum of 3 months following the initial reliable diagnosis.  A high proportion of HOA patients meeting the quality indicator’s requirements may indicate that exercise therapy as a core element of osteoarthritis treatment was initiated at an early stage of disease. By this, strengthening of the muscles surrounding the joint is initiated and carried out correctly under professional guidance. |
| **Rationale** | Exercise therapy has a positive effect on pain relief, functional improvement and quality of life. HOA patients should therefore start exercise therapy to strengthen the stabilising muscles surrounding the joint as early as possible to help slow down the progression of osteoarthritis. Targeted, individualised exercise therapy is one of the core elements of osteoarthritis therapy and should be professionally guided. |
| **Original wording** | IF an ambulatory homebound patient is newly diagnosed with osteoarthritis of the knee, has no contraindication to exercise, and is physically and mentally able to exercise, THEN a directed or supervised strengthening or aerobic exercise program should be prescribed within 3 months of diagnosis. [Smith KL, et al. 2007, S. W-44] |
| **Indicator variations** | IF an ambulatory NH Resident is newly diagnosed with symptomatic osteoarthritis of the knee and has no contraindication to exercise and is physically and mentally able to exercise, THEN a directed or supervised strengthening or aerobic exercise program should be prescribed within 1 month of diagnosis. [Zingmond, (2009) supplementary material, S. 46] |
| **Dimension of quality** | Process |
| **Risk adjustment** | No risk adjustment |
| **Data source** | Routine data of statutory health insurances |
| **Literature references** | EUMUSC (2012), MacLean CH, et al. (2007), Smith KL, et al. (2007), Saliba D, et al. (2004), Steel N, et al. (2004) |

## QI6 Minimum duration of 6 months between IA corticosteroid injections

| **QI6** | Minimum duration of 6 months between IA corticosteroid injections |
| --- | --- |
| **Description** | If a HOA patient receives multiple IA corticosteroid injections, the interval between the injections should not be shorter than 6 months. |
| **Note on interpretation** | Not meeting the quality indicator’s requirements may be medically justified in individual cases. The assignment of the billing code for the application of IA corticosteroids to the corresponding HOA diagnosis is possible to a limited extent only. The assignment of the relevant billing code for the application of IA corticosteroids may include injections with other substances or punctures of the joint. Health services paid independently by the insured person (e.g. hyaluronic acid injections) are not depicted in routine data. |
| **Numerator** | HOA patients with a maximum time interval of at least 6 months between two applications of IA corticosteroids^1^  *^1^EBM: 02341 and a diagnosis ICD (M16*, diagnostic certainty: G) in the same billing case* |
| **Denominator** | HOA patients with at least two applications of IA corticosteroids^1^ |
| **Exclusion criteria** | - |
| **Quality objective** | *Aim: high value*  The QI represents the proportion of HOA patients who were applied IA corticosteroids more than once with a maximum injection interval of at least 6 months.  A high proportion of HOA patients meeting the quality indicator’s requirements may indicate an increased appropriateness and safety of the pharmacological therapy, as the risk of side effects should be minimised. |
| **Rationale** | Repeated application of IA corticosteroids increases the risk of undesirable side effects, which is why the injections should only be given with sufficiently long time intervals inbetween. |
| **Original wording** | For patients of knee OA with persistent or moderate to severe pain, intra-articular injection of glucocorticoids is recommended for rapid relief of pain in patients with OA, the injection interval should not be shorter than 4 to 6 months. [Zhang Z, et al. (2020), S. 10] |
| **Indicator variations** | If there are inflammatory findings and the other treatment options have failed, intraarticular glucocorticoids can be applied, not more than 3 times-a-year. [TLAR (2018), S. 1322]  In addition, in the event of a flare, or if general measures and analgesics do not give sufficient pain relief, an intra-articular injection of a corticosteroid, e.g., triamcinolone or methylprednisolone, should be considered. Give 20 to 40 mg each time at intervals of 1 to 3 months. [NHG (2008), S. 116] |
| **Dimension of quality** | Process |
| **Risk adjustment** | No risk adjustment |
| **Data source** | Routine data of statutory health insurances |
| **Literature references** | Zhang Z, et al. (2020), TLAR (2018) |

## QI7 Prescription of strong opioids only after weak opioids have proven ineffective

| **QI7** | Prescription of strong opioids only after weak opioids have proven ineffective |
| --- | --- |
| **Description** | If a HOA patient is contraindicated for TJA and has documentation of being prescribed strong opioids, there should be documentation that weak opioids were prescribed first, unless contraindicated. |
| **Note on interpretation** | Not meeting the quality indicator’s requirements may be medically justified in individual cases. The purchase of non-prescription medications is not depicted in routine data. The assignment of the billing code for the prescribed opioid medications to the corresponding HOA diagnosis is possible to a limited extent only. The decision for TJA depends in particular on the clinical symptoms and the patient's preferences. Only contraindications that can be depicted in routine data can be taken into account. |
| **Numerator** | HOA patients with diagnosis ICD (M16*, diagnostic certainty: G) and contraindication for TJA^1^ and at least one prescription of weak opioids^2^ in the same billing quarter or the billing quarter before the first prescription of strong opioids^3^  *^1^M2Q inpatient and outpatient diagnosis ICD: (5. position: 5=hip & 6=knee):*  *M01.0 Meningococcal arthritis*  *M01.1 Tuberculous arthritis*  *M01.2 Arthritis in Lyme disease*  *M01.3 Arthritis in other bacterial diseases classified elsewhere*  *M01.4 Rubella arthritis*  *M01.5 Arthritis in other viral diseases classified elsewhere*  *M01.6 Arthritis in mycoses*  *M01.8 Arthritis in other infectious and parasitic diseases classified elsewhere*  *M70.5 Other bursitis of knee*  *M70.6 Trochanteric bursitis*  *M70.7 Other bursitis of hip*  *M70.8 Other soft tissue disorders related to use, overuse and pressure*  *M70.9 Unspecified soft tissue disorder related to use, overuse and pressure*  *M73.0* Gonococcal bursitis*  *M73.1* Syphilitic bursitis*  *M73.8* Other soft tissue disorders in diseases classified elsewhere*  *M86.0 Acute haematogenous osteomyelitis*  *M86.1 Other acute osteomyelitis*  *K25*-28* Ulcer in the stomach or duodenum*  *I20*-24* active ischemic heart disease*  *D66 Haemophilia*  *I70.2 Atherosclerosis of arteries of extremities*  *C41* Malignant neoplasm of bone and articular cartilage of other and unspecified localisation*  *G83.1* Monoparesis and monoplegia of a lower limb*  *^2^ATC: N02AA08, N02AA58, N02AX01, N02AX51, N02AX02, N02AA59, N02AA79, N02AJ01 – N02AJ09, N02AJ13 – N02AJ16*  *^3^ATC: N02AA01 – N02AA07, N02AA10, N02AA11, N02AA51 – N02AA56, N02AB*, N02AC*, N02AD*, N02AE*, N02AF*, N02AG*, N02AJ17 – N02AJ19, N02AX03, N02AX05 – N02AX07* |
| **Denominator** | HOA patients with diagnosis ICD (M16*, diagnostic certainty: G) with an initial prescription of strong opioids^3^ |
| **Exclusion criteria** | - |
| **Quality objective** | *Aim: high value*  The QI represents the proportion of HOA patients with a contraindication for TJA and at least one prescription of strong opioids who were prescribed weak opioids before the prescription of strong opioids.  A high proportion of HOA patients meeting the quality indicator’s requirements may indicate an increased appropriateness and continuity care, as the consideration of patient characteristics supports the coordination of individualised osteoarthritis therapy with as few side effects as possible. The therapy should be geared in particular to the main treatment goals of pain reduction and improvement of functionality. |
| **Rationale** | In end-stage osteoarthritis, strong opioids can be prescribed if there is a contraindication to surgery for the HOA patient. The administration of opioids should be based on the guideline on the long-term use of opioids for chronic non-tumour-related pain (LONTS). |
| **Original wording** | Strong opioids must only be prescribed to patients who have a contraindication to knee surgery, who have failed or have a contraindication to other treatments, while taking into account comorbidities and after providing information about adverse effects. [SFR (2020), S. 9] |
| **Indicator variations** | - |
| **Dimension of quality** | Process |
| **Risk adjustment** | No risk adjustment |
| **Data source** | Routine data of statutory health insurances |
| **Literature references** | SFR (2020), ESCEO (2019) |

## QI8 TJA after combined non-pharmacological and pharmacological treatment

| **QI8** | TJA after combined non-pharmacological and pharmacological treatment |
| --- | --- |
| **Description** | If a HOA patient has undergone TJA, there should be documentation of a combination of non-pharmacological and pharmacological treatment modalities before surgery. |
| **Note on interpretation** | Not meeting the quality indicator’s requirements may be medically justified in individual cases. The assignment of the conservative treatment option to the corresponding HOA diagnosis is possible to a limited extent only. The decision for TJA depends in particular on the clinical symptoms and the patient's preferences. |
| **Numerator** | HOA patients with diagnosis ICD (M16*, diagnostic certainty: G) who were prescribed at least one prescription medication^1^ and at least one non-pharmacological prescription therapy^2^ within the year prior to the performed TJA^3^  *^1^ATC: M01*, M02*, N02**  *^2^relevant billing codes for prescription therapies: X03XX – X06XX and X09XX, X10XX except X0607, outpatient rehabilitation: TFA: T07C, course fees: TFA: T047, functional training: EBM: 01621*  *^3^OPS: 5-820** |
| **Denominator** | HOA patients with a diagnosis ICD (M16*, diagnostic certainty: G) and performed TJA^3^ |
| **Exclusion criteria** | - |
| **Quality objective** | *Aim: high value*  The QI represents the proportion of HOA patients who have received a combination of non-pharmacological and pharmacological therapy options before undergoing TJA.  A high proportion of HOA patients meeting the quality indicator’s requirements may indicate an increased appropriateness and continuity of care, as all suitable conservative methods should always precede surgical treatment. |
| **Rationale** | A combination of non-pharmacological and pharmalogical treatment options is considered the gold standard of conservative osteoarthritis treatment and should always precede surgical treatment. |
| **Original wording** | Vor der Indikationsstellung zur Hüft-TEP sollen Patient*innen mit symptomatischer Coxarthrose mit einer Kombination aus medikamentöser und nicht-medikamentöser konservativer Therapie behandelt werden. [AWMF (2021), S. 48] |
| **Indicator variations** | - |
| **Dimension of quality** | Process |
| **Risk adjustment** | No risk adjustment |
| **Data source** | Routine data of statutory health insurances |
| **Literature references** | AWMF (2021), Yeap SS, et al. (2021), Zhang Z, et al. (2020), Yabuki S, et al. (2019), NICE (2020), TLAR (2018), EULAR (2003) |

## QI9 Minimum duration of 3 months of combined non-pharmacological and pharmacological treatment prior to TJA

| **QI9** | Minimum duration of 3 months of combined non-pharmacological and pharmacological treatment prior to TJA |
| --- | --- |
| **Description** | If a HOA patient has undergone TJA, there should be documentation that a combination of non-pharmacological and pharmacological treatment modalities were provided for at least 3 months before surgery. |
| **Note on interpretation** | Not meeting the quality indicator’s requirements may be medically justified in individual cases. The assignment of the conservative treatment option to the corresponding HOA diagnosis is possible to a limited extent only. The decision for TJA depends in particular on the clinical symptoms and the patient's preferences. |
| **Numerator** | HOA patients with initial diagnosis ICD (M16*, diagnostic certainty: G) and a performed TJA^1^  and a time interval of at least 3 months between initial diagnosis and TJA  and at least one prescription of pharmacological therapy options prior to TJA^2^  and at least one prescription of non-pharmacological therapy options prior to TJA^3^  *^1^OPS: 5-820**  *^2^ATC: M01*, M02*, N02**  *^3^relevant billing codes for prescription therapies: X03XX – X06XX and X09XX, X10XX except X0607, outpatient rehabilitation: TFA: T07C, course fees: TFA: T047, functional training: EBM: 01621* |
| **Denominator** | HOA patients with initial diagnosis ICD (M16*, diagnostic certainty: G) and a performed TJA^1^ |
| **Exclusion criteria** | - |
| **Quality objective** | *Aim: high value*  The QI represents the proportion of HOA patients who have undergone TJA at least 3 months after the initial diagnosis and who have received at least one prescription medication and at least one prescription for exercise therapy prior to TJA.  A high proportion of HOA patients meeting the quality indicator’s requirements may indicate an increased appropriateness and continuity of treatment, as the indication for TJA should not be given before the suitable – depending on patient characteristics – conservative treatment alternatives have been applied for a minimum period of time. |
| **Rationale** | As the effects of targeted exercise therapy are limited in time and longer-term drug therapy is associated with relevant complications, the (expected) effectiveness of non-pharmacological and pharmacological therapy must be assessed on the basis of a patient's individual risk-benefit profile. A conservative treatment trial lasting at least 3 months seems reasonable in order to utilise the possible individual benefits of exercise therapy and to reduce the risk of side effects of long-term medication. |
| **Original wording** | Wenn trotz leitliniengerechter konservativer Therapiemaßnahmen über mindestens drei Monate die Patient*innen über einen hohen subjektiven Leidensdruck berichten, sollte die Indikationsstellung zur Hüft-TEP erfolgen. [AWMF (2021), S. 55] |
| **Indicator variations** | - |
| **Dimension of quality** | Process |
| **Risk adjustment** | No risk adjustment |
| **Data source** | Routine data of statutory health insurances |
| **Literature references** | AWMF (2021) |

## QI10 Minimum duration of 3 months between the last IA corticosteroid injection and TJA

| **QI10** | Minimum duration of 3 months between the last IA corticosteroid injection and TJA |
| --- | --- |
| **Description** | If a HOA patient is treated with IA corticosteroids, TJA should not be performed sooner than 3 months after the last IA corticosteroid injection. |
| **Note on interpretation** | Not meeting the quality indicator’s requirements may be medically justified in individual cases. The assignment of the billing code for the application of IA corticosteroids to the corresponding HOA diagnosis is possible to a limited extent only. The relevant billing code may include injections with other substances or punctures of the joint. Health services paid directly by the insured person (e.g. hyaluronic acid injections) are not depicted in routine data. The decision for TJA depends in particular on the clinical symptoms and the patient's preferences. |
| **Numerator** | HOA patients with an application of IA corticosteroids^1^ at least 3 months prior to TJA^2^  *^1^EBM: 02341 and a diagnosis ICD (M16*, diagnostic certainty: G) in the same billing case*  *^2^OPS: Cox.: 5-820** |
| **Denominator** | HOA patients with at least one application of IA corticosteroids^1^ and performed TJA^2^ |
| **Exclusion criteria** | - |
| **Quality objective** | *Aim: high value*  The QI represents the proportion of HOA patients with at least one application of IA corticosteroids and performed TJA whereby the IA corticosteroids were applied at least 3 months prior to TJA.  A high proportion of HOA patients meeting the quality indicator’s requirements may indicate an increased appropriateness and safety of drug therapy, as the risk of periprosthetic complications should be minimised. |
| **Rationale** | The risk of periprosthetic infection is increased if IA corticosteroids were applied within 3 months prior to TJA. However, the exact period in which there is an increased risk of infection can also be shorter than 3 months, particularly in the case of very severe suffering or very severe restrictions. The highest possible patient safety is guaranteed if TJA is performed at the earliest 6 weeks after an injection with IA corticosteroids, but 3 months is recommended. 3 months is also considered a reasonable waiting time before surgery. |
| **Original wording** | Nach einer intraartikulären Injektion von Kortikosteroiden sollte eine Hüft-Endoprothesenoperation frühestens nach 6 Wochen erfolgen, zu empfehlen jedoch erst nach 3 Monaten. [AWMF (2021), S. 83] |
| **Indicator variations** | - |
| **Dimension of quality** | Process |
| **Risk adjustment** | No risk adjustment |
| **Data source** | Routine data of statutory health insurances |
| **Literature references** | AWMF (2021) |

## QI11 Specialist physician visit only with referral

| **QI11** | Specialist physician visit only with referral |
| --- | --- |
| **Description** | If a HOA patient has an appointment with a physician providing outpatient specialist care, there should be documentation of a prior referral. |
| **Note on interpretation** | Not meeting the quality indicator’s requirements may be medically justified in individual cases or are owed to the patient’s interests or behaviour. The patient’s utilisation of health services cannot be completely controlled by medical service providers. The coding of referrals in routine data is not always given, so that the indicator may not capture all referrals that are made in the reality of care. |
| **Numerator** | Number of HOA patients’ specialist visits^1^ with preceding referral  ^1^billing contact at specialists for internal medicine and rheumatology, orthopaedics, orthopaedics and trauma surgery, physical and rehabilitative medicine, radiology with a HOA diagnosis (M16*, diagnostic certainty: G) |
| **Denominator** | Number of HOA patients’ appointments at specialist physicians^1^ |
| **Exclusion criteria** | - |
| **Quality objective** | *Aim: high value*  The QI represents the proportion of HOA patients for whom a referral precedes the billing contact with a specialist physician.  A high proportion of HOA patients meeting the quality indicator’s requirements may indicate an increased coordination of care. |
| **Rationale** | Health care for HOA patients should be coordinated. The medical services utilised should be managed by a central provider, usually a general practitioner. This helps to ensure that patients receive appropriate and sequential treatment. |
| **Original wording** | - |
| **Indicator variations** | - |
| **Dimension of quality** | Process |
| **Risk adjustment** | No risk adjustment |
| **Data source** | Routine data of statutory health insurances |
| **Literature references** | (based on Delphi panel consensus) |

## QI12 Orthopaedic surgeon visit only with referral

| **QI12** | Orthopaedic surgeon visit only with referral |
| --- | --- |
| **Description** | If a HOA patient has an appointment with an orthopaedic surgeon, there should be documentation of a prior referral. |
| **Note on interpretation** | Not meeting the quality indicator’s requirements may be medically justified in individual cases or are owed to the patient’s interests or behaviour. The patient’s utilisation of health services cannot be completely controlled by medical service providers. The coding of referrals in routine data is not always given, so that the indicator may not capture all referrals that are made in the reality of care. |
| **Numerator** | Number of HOA patients’ specialist visits^1^ with preceding referral  ^1^billing contact at specialists for orthopaedics, orthopaedics and trauma surgery with HOA diagnosis (M16*, diagnostic certainty: G) |
| **Denominator** | Number of HOA patients’ appointments at specialist physicians^1^ |
| **Exclusion criteria** | - |
| **Quality objective** | *Aim: high value*  The QI represents the proportion of HOA patients for whom a referral precedes the billing contact with an orthopaedic surgeon.  A high proportion of HOA patients meeting the quality indicator’s requirements may indicate an increased coordination and continuity care. |
| **Rationale** | TJA is an effective therapy option to improve functionality and relieve pain and should therefore be performed after conservative therapy has proven ineffective. If symptoms persist despite conservative therapy, a referral should be made to an orthopaedic surgeon so that the necessity and chances of success of TJA can be assessed. |
| **Original wording** | If a patient is diagnosed with OA and has been referred to an orthopedic surgeon, then the waiting time from first referral should not exceed three months. [EUMUSC.net (2012)] |
| **Indicator variations** | - |
| **Dimension of quality** | Process |
| **Risk adjustment** | No risk adjustment |
| **Data source** | Routine data of statutory health insurances |
| **Literature references** | EUMUSC (2012) |

# OSTEOARTHRITIS OF THE KNEE

## QI1 Initial diagnosis using radiographic examination

| **QI1** | Initial diagnosis using radiographic examination |
| --- | --- |
| **Description** | If a patient is newly diagnosed with KOA, the diagnosis should be based on radiographic imaging. |
| **Note on interpretation** | Not meeting the quality indicator’s requirements may be medically justified in individual cases. The assignment of the billing code for the radiographic examination to the corresponding KOA diagnosis is possible to a limited extent only. In addition, the functional limitations and clinical complaints of the patient are decisive for a treatment decision. |
| **Numerator** | KOA patients with a billing code for a radiographic examination^1^ in the same, the preceding or the following billing quarter as the initial diagnosis  *^1^EBM: 34233, 34235 and diagnosis ICD (M17*,* *diagnostic certainty: G) in the same billing case* |
| **Denominator** | KOA patients with initial diagnosis ICD (M17*, diagnostic certainty: G) |
| **Exclusion criteria** | - |
| **Quality objective** | *Aim: high value*  The QI represents the proportion of KOA patients with radiographic examination used for determining the initial diagnosis.  A high proportion of KOA patients meeting the quality indicator’s requirements may indicate an increased treatment safety, as a reliable diagnosis ensures targeted and needs-based coordination of medical care. |
| **Rationale** | In addition to the clinical examination, the radiographic examination is used to classify the progression of the osteoarthritis disease. This can lead to an improved treatment outcome and progress monitoring. |
| **Original wording** | Die Diagnose der Gonarthrose soll klinisch und radiologisch gestellt werden. [AWMF (2018), S. 8] |
| **Indicator variations** | - |
| **Dimension of quality** | Process |
| **Risk adjustment** | No risk adjustment |
| **Data source** | Routine data of statutory health insurances |
| **Literature references** | AWMF (2019), AWMF (2018) |

## QI2 Radiography as primary imaging diagnostics

| **QI2** | Radiography as primary imaging diagnostics |
| --- | --- |
| **Description** | If the initial diagnosis of KOA was based on imaging, conventional radiography should have been used before other imaging modalities (MRI, CT, sonography). |
| **Note on interpretation** | Not meeting the quality indicator’s requirements may be medically justified in individual cases. The assignment of the billing code for the radiographic examination to the corresponding KOA diagnosis is possible to a limited extent only. |
| **Numerator** | KOA patients with a billing code for a radiographic examination^1^ in the same, the preceding or the following billing quarter as the initial diagnosis  *^1^EBM: 34233, 34235 and diagnosis ICD (M17*,* *diagnostic certainty: G) in the same billing case* |
| **Denominator** | KOA patients with a billing code for a radiologic examination (radiography, MRI, CT, sonography)^2^ in the same, the preceding or the following billing quarter as the initial diagnosis  *^2^EBM: 34233, 34235, 33050, 34350, 34450 and diagnosis ICD (M17*,* *diagnostic certainty: G) in the same billing case* |
| **Exclusion criteria** | - |
| **Quality objective** | *Aim: high value*  The QI represents the proportion of KOA patients for whom the radiological diagnosis was first made by radiographic examination before other imaging procedures (sonography, CT, MRI) were considered.  A high proportion of KOA patients meeting the quality indicator’s requirements may indicate an increased appropriateness, as other imaging procedures (sonography, CT, MRI) should only be performed in cases of unclear joint symptoms, discrepancy between clinical and radiographic findings or lack of therapeutic success. |
| **Rationale** | Radiographic examination is an inexpensive and easily performable procedure for assessing the severity of osteoarthritis and the associated possible surgical procedures and expected complications. |
| **Original wording** | If imaging is needed, conventional (plain) radiography should be used before other modalities. To make additional diagnoses, soft tissues are best imaged by US or MRI and bone by CT or MRI. [EULAR (2017) S. 1486] |
| **Indicator variations** | - |
| **Dimension of quality** | Process |
| **Risk adjustment** | No risk adjustment |
| **Data source** | Routine data of statutory health insurances |
| **Literature references** | AWMF (2021), SIR (2019), AWMF (2019), AWMF (2018), EULAR (2017), NHG (2008) |

## QI3a Number of radiographic examinations performed per patient

| **QI3a** | Number of radiographic examinations performed per patient |
| --- | --- |
| **Description** | Radiographic examination in KOA patients should be performed only as medically necessary. |
| **Note on interpretation** | Not meeting the quality indicator’s requirements may be medically justified in individual cases. The assignment of the billing code for the radiographic examination to the corresponding KOA diagnosis is possible to a limited extent only. Once there is a reliable diagnosis, treatment decisions should be based primarily on the clinical examination, not the radiological examination. |
| **Numerator** | Number of performed radiographic examinations^1^  *^1^EBM: 34233, 34235 and diagnosis ICD (M17*,* *diagnostic certainty: G) in the same billing case* |
| **Denominator** | Number of KOA patients with at least one radiographic examination^1^ |
| **Exclusion criteria** | - |
| **Quality objective** | *Aim: low value*  The QI represents the number of radiographic examinations performed per KOA patient (with at least one radiographic examination performed).  A high number of radiographic examinations performed per KOA patient compared to other health service providers may indicate inadequate coordination or overuse. |
| **Rationale** | Radiological imaging in KOA patients should be performed as needed and appropriate. A high rate of radiographic examinations per patient may be an indication of overprovision. |
| **Original wording** | - |
| **Indicator variations** | - |
| **Dimension of quality** | Process |
| **Risk adjustment** | No risk adjustment |
| **Data source** | Routine data of statutory health insurances |
| **Literature references** | (based on Delphi panel consensus) |

## QI3b Number of MRI examinations performed per patient

| **QI3b** | Number of MRI examinations performed per patient |
| --- | --- |
| **Description** | MRI examinations in KOA patients should be performed only as medically necessary. |
| **Note on interpretation** | Not meeting the quality indicator’s requirements may be medically justified in individual cases. The assignment of the billing code for the MRI examination to the corresponding KOA diagnosis is possible to a limited extent only. Once there is a reliable diagnosis, treatment decisions should be based primarily on the clinical examination, not the radiological examination. |
| **Numerator** | Number of performed MRI examinations^1^  *^1^EBM: 34450 and diagnosis ICD (M17*,* *diagnostic certainty: G) in the same billing case* |
| **Denominator** | Number of KOA patients with at least one MRI examination^1^ |
| **Exclusion criteria** | - |
| **Quality objective** | *Aim: low value*  The QI represents the number of MRI examinations performed per KOA patient (with at least one MRI examination performed).  A high number of MRI examinations performed per KOA patient compared to other health service providers may indicate inadequate coordination or overuse. |
| **Rationale** | Radiological imaging in KOA patients should be performed as needed and appropriate. A high rate of MRI examinations per patient may be an indication of overprovision. |
| **Original wording** | - |
| **Indicator variations** | - |
| **Dimension of quality** | Process |
| **Risk adjustment** | No risk adjustment |
| **Data source** | Routine data of statutory health insurances |
| **Literature references** | (based on Delphi panel consensus) |

## QI3c Number of CT examinations performed per patient

| **QI3c** | Number of CT examinations performed per patient |
| --- | --- |
| **Description** | CT examinations in KOA patients should be performed only as medically necessary. |
| **Note on interpretation** | Not meeting the quality indicator’s requirements may be medically justified in individual cases. The assignment of the billing code for the CT examination to the corresponding KOA diagnosis is possible to a limited extent only. Once there is a reliable diagnosis, treatment decisions should be based primarily on the clinical examination, not the radiological examination. |
| **Numerator** | Number of performed CT examinations^1^  *^1^EBM: 34350 and diagnosis ICD (M17*,* *diagnostic certainty: G) in the same billing case* |
| **Denominator** | Number of KOA patients with at least one CT examination^1^ |
| **Exclusion criteria** | - |
| **Quality objective** | *Aim: low value*  The QI represents the number of CT examinations performed per KOA patient (with at least one CT examination performed).  A high number of CT examinations performed per KOA patient compared to other health service providers may indicate inadequate coordination or overuse. |
| **Rationale** | Radiological imaging in KOA patients should be performed as needed and appropriate. A high rate of CT examinations per patient may be an indication of overprovision. |
| **Original wording** | - |
| **Indicator variations** | - |
| **Dimension of quality** | Process |
| **Risk adjustment** | No risk adjustment |
| **Data source** | Routine data of statutory health insurances |
| **Literature references** | (based on Delphi panel consensus) |

## QI3d Number of sonographic examinations performed per patient

| **QI3d** | Number of sonographic examinations performed per patient |
| --- | --- |
| **Description** | Sonographic examinations in KOA patients should be performed only as medically necessary. |
| **Note on interpretation** | Not meeting the quality indicator’s requirements may be medically justified in individual cases. The assignment of the billing code for the sonographic examination to the corresponding KOA diagnosis is possible to a limited extent only. Once there is a reliable diagnosis, treatment decisions should be based primarily on the clinical examination, not the radiological examination. |
| **Numerator** | Number of performed sonographic examinations^1^  *^1^EBM: 33050 and diagnosis ICD (M17*,* *diagnostic certainty: G) in the same billing case* |
| **Denominator** | Number of KOA patients with at least one sonographic examination^1^ |
| **Exclusion criteria** | - |
| **Quality objective** | *Aim: low value*  The QI represents the number of sonographic examinations performed per KOA patient (with at least one sonographic examination performed).  A high number of sonographic examinations performed per KOA patient compared to other health service providers may indicate inadequate coordination or overuse. |
| **Rationale** | Radiological imaging in KOA patients should be performed as needed and appropriate. A high rate of sonographic examinations per patient may be an indication of overprovision. |
| **Original wording** | - |
| **Indicator variations** | - |
| **Dimension of quality** | Process |
| **Risk adjustment** | No risk adjustment |
| **Data source** | Routine data of statutory health insurances |
| **Literature references** | (based on Delphi panel consensus) |

## QI4 Documentation of at least one referral to supervised exercise therapy within 12 months after initial diagnosis

| **QI4** | Documentation of at least one referral to supervised exercise therapy within 12 months after initial diagnosis |
| --- | --- |
| **Description** | If a patient has had a diagnosis of KOA for >12 months, there should be documentation of at least one referral to supervised exercise therapy within 12 months after the initial KOA diagnosis. |
| **Note on interpretation** | Not meeting the quality indicator’s requirements may be medically justified in individual cases. The assignment of the billing code for the radiographic examination to the corresponding KOA diagnosis is possible to a limited extent only. Instructional activities beyond the billing-relevant provision of health services (e.g. instructions for self-management), which also contribute to the quality of care, are not depicted. |
| **Numerator** | KOA patients with initial diagnosis ICD (M17*, diagnostic certainty: G) and at least one prescription for exercise therapy^1^ within 12 months after initial diagnosis  *^1^relevant billing codes for prescription therapies: X03XX – X06XX and X09XX, X10XX except X0607, outpatient rehabilitation: TFA: T07C, course fees: TFA: T047, functional training: EBM: 01621* |
| **Denominator** | KOA patients with initial diagnosis ICD (M17*, diagnostic certainty: G) |
| **Exclusion criteria** | - |
| **Quality objective** | *Aim: high value*  The QI represents the proportion of KOA patients who were referred to supervised exercise therapy at least once within a maximum of 12 months following the initial diagnosis.  A high proportion of KOA patients meeting the quality indicator’s requirements may indicate that supervised exercise therapy as a core element of osteoarthritis treatment was initiated at an early stage of disease. By this, strengthening of the muscles surrounding the joint is initiated and carried out correctly under professional guidance. |
| **Rationale** | Exercise therapy has a positive effect on pain relief, functional improvement and quality of life. KOA patients should therefore start exercise therapy to strengthen the stabilising muscles surrounding the joint as early as possible to help slow down the progression of osteoarthritis. Targeted, individualised exercise therapy is one of the core elements of osteoarthritis therapy and should be professionally guided. |
| **Original wording** | IF an ambulatory NH Resident has had a diagnosis of symptomatic osteoarthritis of the knee for >12 months and has no contraindication to exercise and is physically and mentally able to exercise, THEN there should be evidence that a directed or supervised strengthening or aerobic exercise program was prescribed at least once since the time of diagnosis. [Zingmond DS, et al. (2009), supplementary material, S. 46] |
| **Indicator variations** | IF an ambulatory NH resident has a diagnosis of symptomatic knee osteoarthritis for >3 months, has no contraindication to exercise, and is physically and mentally able to exercise THEN there should be evidence that a directed or supervised strengthening or aerobic exercise program was prescribed at least once since the time of diagnosis. [Saliba D (2004), S. 304]  Providers caring for patients with symptoms of hip or knee OA should recommend exercise programs at least once in 2 years. [McGlynn EA, et al. (2003), Appendix A, S. 39; Asch SM (2004), S. W-200]  Providers caring for patients with symptoms of hip or knee OA should recommend both of the following at least once in 2 years:  a. exercise programs for persons with hip or knee OA;  b. weight loss among persons with knee OA and a BMI >25. [Moore A (2000), S. 260] |
| **Dimension of quality** | Process |
| **Risk adjustment** | No risk adjustment |
| **Data source** | Routine data of statutory health insurances |
| **Literature references** | Doubova SV, et al. (2015), Zingmond DS, et al. (2009), Smith KL, et al. (2007) |

## QI5 Initiation of supervised exercise therapy within 3 months after initial diagnosis

| **QI5** | Initiation of supervised exercise therapy within 3 months after initial diagnosis |
| --- | --- |
| **Description** | If a patient has had a diagnosis of KOA for >3 months, there should be documentation that the first referral to supervised exercise therapy took place within 3 months after the initial KOA diagnosis. |
| **Note on interpretation** | Not meeting the quality indicator’s requirements may be medically justified in individual cases. The assignment of the billing code for the radiographic examination to the corresponding KOA diagnosis is possible to a limited extent only. Advising and informing activities beyond the billing-relevant provision of health services (e.g. instructions for self-management), which also contribute to the quality of care, are not depicted. |
| **Numerator** | KOA patients with initial diagnosis ICD (M17*, diagnostic certainty: G) and at least one prescription for exercise therapy^1^ within 3 months after initial diagnosis  *^1^relevant billing codes for prescription therapies: X03XX – X06XX and X09XX, X10XX except X0607, outpatient rehabilitation: TFA: T07C, course fees: TFA: T047, functional training: EBM: 01621* |
| **Denominator** | KOA patients with initial diagnosis ICD (M17*, diagnostic certainty: G) |
| **Exclusion criteria** | - |
| **Quality objective** | *Aim: high value*  The QI represents the proportion of KOA patients who were referred to supervised exercise therapy at least once within a maximum of 3 months following the initial reliable diagnosis.  A high proportion of KOA patients meeting the quality indicator’s requirements may indicate that exercise therapy as a core element of osteoarthritis treatment was initiated at an early stage of disease. By this, strengthening of the muscles surrounding the joint is initiated and carried out correctly under professional guidance. |
| **Rationale** | Exercise therapy has a positive effect on pain relief, functional improvement and quality of life. KOA patients should therefore start exercise therapy to strengthen the stabilising muscles surrounding the joint as early as possible to help slow down the progression of osteoarthritis. Targeted, individualised exercise therapy is one of the core elements of osteoarthritis therapy and should be professionally guided. |
| **Original wording** | IF an ambulatory homebound patient is newly diagnosed with osteoarthritis of the knee, has no contraindication to exercise, and is physically and mentally able to exercise, THEN a directed or supervised strengthening or aerobic exercise program should be prescribed within 3 months of diagnosis. [Smith KL, et al. 2007, S. W-44] |
| **Indicator variations** | IF an ambulatory NH Resident is newly diagnosed with symptomatic osteoarthritis of the knee and has no contraindication to exercise and is physically and mentally able to exercise, THEN a directed or supervised strengthening or aerobic exercise program should be prescribed within 1 month of diagnosis. [Zingmond, (2009) supplementary material, S. 46] |
| **Dimension of quality** | Process |
| **Risk adjustment** | No risk adjustment |
| **Data source** | Routine data of statutory health insurances |
| **Literature references** | EUMUSC (2012), MacLean CH, et al. (2007), Smith KL, et al. (2007), Saliba D, et al. (2004), Steel N, et al. (2004) |

## QI6 Minimum duration of 6 months between IA corticosteroid injections

| **QI6** | Minimum duration of 6 months between IA corticosteroid injections |
| --- | --- |
| **Description** | If a KOA patient receives multiple IA corticosteroid injections, the interval between the injections should not be shorter than 6 months. |
| **Note on interpretation** | Not meeting the quality indicator’s requirements may be medically justified in individual cases. The assignment of the billing code for the application of IA corticosteroids to the corresponding KOA diagnosis is possible to a limited extent only. The assignment of the relevant billing code for the application of IA corticosteroids may include injections with other substances or punctures of the joint. Health services paid independently by the insured person (e.g. hyaluronic acid injections) are not depicted in routine data. |
| **Numerator** | KOA patients with a maximum time interval of at least 6 months between two applications of IA corticosteroids^1^  *^1^EBM: 02341 and a diagnosis ICD (M17*, diagnostic certainty: G) in the same billing case* |
| **Denominator** | KOA patients with at least two applications of IA corticosteroids^1^ |
| **Exclusion criteria** | - |
| **Quality objective** | *Aim: high value*  The QI represents the proportion of KOA patients who were applied IA corticosteroids more than once with a maximum injection interval of at least 6 months.  A high proportion of KOA patients meeting the quality indicator’s requirements may indicate an increased appropriateness and safety of the pharmacological therapy, as the risk of side effects should be minimised. |
| **Rationale** | Repeated application of IA corticosteroids increases the risk of undesirable side effects, which is why the injections should only be given with sufficiently long time intervals inbetween. |
| **Original wording** | For patients of knee OA with persistent or moderate to severe pain, intra-articular injection of glucocorticoids is recommended for rapid relief of pain in patients with OA, the injection interval should not be shorter than 4 to 6 months. [Zhang Z, et al. (2020), S. 10] |
| **Indicator variations** | If there are inflammatory findings and the other treatment options have failed, intraarticular glucocorticoids can be applied, not more than 3 times-a-year. [TLAR (2018), S. 1322]  In addition, in the event of a flare, or if general measures and analgesics do not give sufficient pain relief, an intra-articular injection of a corticosteroid, e.g., triamcinolone or methylprednisolone, should be considered. Give 20 to 40 mg each time at intervals of 1 to 3 months. [NHG (2008), S. 116] |
| **Dimension of quality** | Process |
| **Risk adjustment** | No risk adjustment |
| **Data source** | Routine data of statutory health insurances |
| **Literature references** | Zhang Z, et al. (2020), TLAR (2018) |

## QI7 Prescription of strong opioids only after weak opioids have proven ineffective

| **QI7** | Prescription of strong opioids only after weak opioids have proven ineffective |
| --- | --- |
| **Description** | If a KOA patient is contraindicated for TJA and has documentation of being prescribed strong opioids, there should be documentation that weak opioids were prescribed first, unless contraindicated. |
| **Note on interpretation** | Not meeting the quality indicator’s requirements may be medically justified in individual cases. The purchase of non-prescription medications is not depicted in routine data. The assignment of the billing code for the prescribed opioid medications to the corresponding KOA diagnosis is possible to a limited extent only. The decision for TJA depends in particular on the clinical symptoms and the patient's preferences. Only contraindications that can be depicted in routine data can be taken into account. |
| **Numerator** | KOA patients with diagnosis ICD (M17*, diagnostic certainty: G) and contraindication for TJA^1^ and at least one prescription of weak opioids^2^ in the same billing quarter or the billing quarter before the first prescription of strong opioids^3^  *^1^M2Q inpatient and outpatient diagnosis ICD: (5. position: 5=hip & 6=knee):*  *M01.0 Meningococcal arthritis*  *M01.1 Tuberculous arthritis*  *M01.2 Arthritis in Lyme disease*  *M01.3 Arthritis in other bacterial diseases classified elsewhere*  *M01.4 Rubella arthritis*  *M01.5 Arthritis in other viral diseases classified elsewhere*  *M01.6 Arthritis in mycoses*  *M01.8 Arthritis in other infectious and parasitic diseases classified elsewhere*  *M70.5 Other bursitis of knee*  *M70.6 Trochanteric bursitis*  *M70.7 Other bursitis of hip*  *M70.8 Other soft tissue disorders related to use, overuse and pressure*  *M70.9 Unspecified soft tissue disorder related to use, overuse and pressure*  *M73.0* Gonococcal bursitis*  *M73.1* Syphilitic bursitis*  *M73.8* Other soft tissue disorders in diseases classified elsewhere*  *M86.0 Acute haematogenous osteomyelitis*  *M86.1 Other acute osteomyelitis*  *K25*-28* Ulcer in the stomach or duodenum*  *I20*-24* active ischemic heart disease*  *D66 Haemophilia*  *I70.2 Atherosclerosis of arteries of extremities*  *C41* Malignant neoplasm of bone and articular cartilage of other and unspecified localisation*  *G83.1* Monoparesis and monoplegia of a lower limb*  *^2^ATC: N02AA08, N02AA58, N02AX01, N02AX51, N02AX02, N02AA59, N02AA79, N02AJ01 – N02AJ09, N02AJ13 – N02AJ16*  *^3^ATC: N02AA01 – N02AA07, N02AA10, N02AA11, N02AA51 – N02AA56, N02AB*, N02AC*, N02AD*, N02AE*, N02AF*, N02AG*, N02AJ17 – N02AJ19, N02AX03, N02AX05 – N02AX07* |
| **Denominator** | KOA patients with diagnosis ICD (M17*, diagnostic certainty: G) with an initial prescription of strong opioids^3^ |
| **Exclusion criteria** | - |
| **Quality objective** | *Aim: high value*  The QI represents the proportion of KOA patients with a contraindication for TJA and at least one prescription of strong opioids who were prescribed weak opioids before the prescription of strong opioids.  A high proportion of KOA patients meeting the quality indicator’s requirements may indicate an increased appropriateness and continuity care, as the consideration of patient characteristics supports the coordination of individualised osteoarthritis therapy with as few side effects as possible. The therapy should be geared in particular to the main treatment goals of pain reduction and improvement of functionality. |
| **Rationale** | In end-stage osteoarthritis, strong opioids can be prescribed if there is a contraindication to surgery for the KOA patient. The administration of opioids should be based on the guideline on the long-term use of opioids for chronic non-tumour-related pain (LONTS). |
| **Original wording** | Strong opioids must only be prescribed to patients who have a contraindication to knee surgery, who have failed or have a contraindication to other treatments, while taking into account comorbidities and after providing information about adverse effects. [SFR (2020), S. 9] |
| **Indicator variations** | - |
| **Dimension of quality** | Process |
| **Risk adjustment** | No risk adjustment |
| **Data source** | Routine data of statutory health insurances |
| **Literature references** | SFR (2020), ESCEO (2019) |

## QI8 TJA after combined non-pharmacological and pharmacological treatment

| **QI8** | TJA after combined non-pharmacological and pharmacological treatment |
| --- | --- |
| **Description** | If a KOA patient has undergone TJA, there should be documentation of a combination of non-pharmacological and pharmacological treatment modalities before surgery. |
| **Note on interpretation** | Not meeting the quality indicator’s requirements may be medically justified in individual cases. The assignment of the conservative treatment option to the corresponding KOA diagnosis is possible to a limited extent only. The decision for TJA depends in particular on the clinical symptoms and the patient's preferences. |
| **Numerator** | KOA patients with diagnosis ICD (M17*, diagnostic certainty: G) who were prescribed at least one prescription medication^1^ and at least one non-pharmacological prescription therapy^2^ within the year prior to the performed TJA^3^  *^1^ATC: M01*, M02*, N02**  *^2^relevant billing codes for prescription therapies: X03XX – X06XX and X09XX, X10XX except X0607, outpatient rehabilitation: TFA: T07C, course fees: TFA: T047, functional training: EBM: 01621*  *^3^OPS: 5-822** |
| **Denominator** | KOA patients with a diagnosis ICD (M17*, diagnostic certainty: G) and performed TJA^3^ |
| **Exclusion criteria** | - |
| **Quality objective** | *Aim: high value*  The QI represents the proportion of KOA patients who have received a combination of non-pharmacological and pharmacological therapy options before undergoing TJA.  A high proportion of KOA patients meeting the quality indicator’s requirements may indicate an increased appropriateness and continuity of care, as all suitable conservative methods should always precede surgical treatment. |
| **Rationale** | A combination of non-pharmacological and pharmalogical treatment options is considered the gold standard of conservative osteoarthritis treatment and should always precede surgical treatment. |
| **Original wording** | Vor der Indikationsstellung zur Hüft-TEP sollen Patient*innen mit symptomatischer Coxarthrose mit einer Kombination aus medikamentöser und nicht-medikamentöser konservativer Therapie behandelt werden. [AWMF (2021), S. 48] |
| **Indicator variations** | - |
| **Dimension of quality** | Process |
| **Risk adjustment** | No risk adjustment |
| **Data source** | Routine data of statutory health insurances |
| **Literature references** | AWMF (2021), Yeap SS, et al. (2021), Zhang Z, et al. (2020), Yabuki S, et al. (2019), NICE (2020), TLAR (2018), EULAR (2003) |

## QI9 Minimum duration of 3 months of combined non-pharmacological and pharmacological treatment prior to TJA

| **QI9** | Minimum duration of 3 months of combined non-pharmacological and pharmacological treatment prior to TJA |
| --- | --- |
| **Description** | If a KOA patient has undergone TJA, there should be documentation that a combination of non-pharmacological and pharmacological treatment modalities were provided for at least 3 months before surgery. |
| **Note on interpretation** | Not meeting the quality indicator’s requirements may be medically justified in individual cases. The assignment of the conservative treatment option to the corresponding KOA diagnosis is possible to a limited extent only. The decision for TJA depends in particular on the clinical symptoms and the patient's preferences. |
| **Numerator** | KOA patients with initial diagnosis ICD (M17*, diagnostic certainty: G) and a performed TJA^1^  and a time interval of at least 3 months between initial diagnosis and TJA  and at least one prescription of pharmacological therapy options prior to TJA^2^  and at least one prescription of non-pharmacological therapy options prior to TJA^3^  *^1^OPS: 5-822**  *^2^ATC: M01*, M02*, N02**  *^3^relevant billing codes for prescription therapies: X03XX – X06XX and X09XX, X10XX except X0607, outpatient rehabilitation: TFA: T07C, course fees: TFA: T047, functional training: EBM: 01621* |
| **Denominator** | KOA patients with initial diagnosis ICD (M17*, diagnostic certainty: G) and a performed TJA^1^ |
| **Exclusion criteria** | - |
| **Quality objective** | *Aim: high value*  The QI represents the proportion of KOA patients who have undergone TJA at least 3 months after the initial diagnosis and who have received at least one prescription medication and at least one prescription for exercise therapy prior to TJA.  A high proportion of KOA patients meeting the quality indicator’s requirements may indicate an increased appropriateness and continuity of treatment, as the indication for TJA should not be given before the suitable – depending on patient characteristics – conservative treatment alternatives have been applied for a minimum period of time. |
| **Rationale** | As the effects of targeted exercise therapy are limited in time and longer-term drug therapy is associated with relevant complications, the (expected) effectiveness of non-pharmacological and pharmacological therapy must be assessed on the basis of a patient's individual risk-benefit profile. A conservative treatment trial lasting at least 3 months seems reasonable in order to utilise the possible individual benefits of exercise therapy and to reduce the risk of side effects of long-term medication. |
| **Original wording** | Wenn trotz leitliniengerechter konservativer Therapiemaßnahmen über mindestens drei Monate die Patient*innen über einen hohen subjektiven Leidensdruck berichten, sollte die Indikationsstellung zur Hüft-TEP erfolgen. [AWMF (2021), S. 55] |
| **Indicator variations** | - |
| **Dimension of quality** | Process |
| **Risk adjustment** | No risk adjustment |
| **Data source** | Routine data of statutory health insurances |
| **Literature references** | AWMF (2021) |

## QI10 Minimum duration of 3 months between the last IA corticosteroid injection and TJA

| **QI10** | Minimum duration of 3 months between the last IA corticosteroid injection and TJA |
| --- | --- |
| **Description** | If a KOA patient is treated with IA corticosteroids, TJA should not be performed sooner than 3 months after the last IA corticosteroid injection. |
| **Note on interpretation** | Not meeting the quality indicator’s requirements may be medically justified in individual cases. The assignment of the billing code for the application of IA corticosteroids to the corresponding KOA diagnosis is possible to a limited extent only. The relevant billing code may include injections with other substances or punctures of the joint. Health services paid directly by the insured person (e.g. hyaluronic acid injections) are not depicted in routine data. The decision for TJA depends in particular on the clinical symptoms and the patient's preferences. |
| **Numerator** | KOA patients with an application of IA corticosteroids^1^ at least 3 months prior to TJA^2^  *^1^EBM: 02341 and a diagnosis ICD (M17*, diagnostic certainty: G) in the same billing case*  *^2^OPS: Cox.: 5-822** |
| **Denominator** | KOA patients with at least one application of IA corticosteroids^1^ and performed TJA^2^ |
| **Exclusion criteria** | - |
| **Quality objective** | *Aim: high value*  The QI represents the proportion of KOA patients with at least one application of IA corticosteroids and performed TJA whereby the IA corticosteroids were applied at least 3 months prior to TJA.  A high proportion of KOA patients meeting the quality indicator’s requirements may indicate an increased appropriateness and safety of drug therapy, as the risk of periprosthetic complications should be minimised. |
| **Rationale** | The risk of periprosthetic infection is increased if IA corticosteroids were applied within 3 months prior to TJA. However, the exact period in which there is an increased risk of infection can also be shorter than 3 months, particularly in the case of very severe suffering or very severe restrictions. The highest possible patient safety is guaranteed if TJA is performed at the earliest 6 weeks after an injection with IA corticosteroids, but 3 months is recommended. 3 months is also considered a reasonable waiting time before surgery. |
| **Original wording** | Nach einer intraartikulären Injektion von Kortikosteroiden sollte eine Hüft-Endoprothesenoperation frühestens nach 6 Wochen erfolgen, zu empfehlen jedoch erst nach 3 Monaten. [AWMF (2021), S. 83] |
| **Indicator variations** | - |
| **Dimension of quality** | Process |
| **Risk adjustment** | No risk adjustment |
| **Data source** | Routine data of statutory health insurances |
| **Literature references** | AWMF (2021) |

## QI11 Specialist physician visit only with referral

| **QI11** | Specialist physician visit only with referral |
| --- | --- |
| **Description** | If a KOA patient has an appointment with a physician providing outpatient specialist care, there should be documentation of a prior referral. |
| **Note on interpretation** | Not meeting the quality indicator’s requirements may be medically justified in individual cases or are owed to the patient’s interests or behaviour. The patient’s utilisation of health services cannot be completely controlled by medical service providers. The coding of referrals in routine data is not always given, so that the indicator may not capture all referrals that are made in the reality of care. |
| **Numerator** | Number of KOA patients’ specialist visits^1^ with preceding referral  ^1^billing contact at specialists for internal medicine and rheumatology, orthopaedics, orthopaedics and trauma surgery, physical and rehabilitative medicine, radiology with a KOA diagnosis (M17*, diagnostic certainty: G) |
| **Denominator** | Number of KOA patients’ appointments at specialist physicians^1^ |
| **Exclusion criteria** | - |
| **Quality objective** | *Aim: high value*  The QI represents the proportion of KOA patients for whom a referral precedes the billing contact with a specialist physician.  A high proportion of KOA patients meeting the quality indicator’s requirements may indicate an increased coordination of care. |
| **Rationale** | Health care for KOA patients should be coordinated. The medical services utilised should be managed by a central provider, usually a general practitioner. This helps to ensure that patients receive appropriate and sequential treatment. |
| **Original wording** | - |
| **Indicator variations** | - |
| **Dimension of quality** | Process |
| **Risk adjustment** | No risk adjustment |
| **Data source** | Routine data of statutory health insurances |
| **Literature references** | (based on Delphi panel consensus) |

## QI12 Orthopaedic surgeon visit only with referral

| **QI12** | Orthopaedic surgeon visit only with referral |
| --- | --- |
| **Description** | If a KOA patient has an appointment with an orthopaedic surgeon, there should be documentation of a prior referral. |
| **Note on interpretation** | Not meeting the quality indicator’s requirements may be medically justified in individual cases or are owed to the patient’s interests or behaviour. The patient’s utilisation of health services cannot be completely controlled by medical service providers. The coding of referrals in routine data is not always given, so that the indicator may not capture all referrals that are made in the reality of care. |
| **Numerator** | Number of KOA patients’ specialist visits^1^ with preceding referral  ^1^billing contact at specialists for orthopaedics, orthopaedics and trauma surgery with KOA diagnosis (M17*, diagnostic certainty: G) |
| **Denominator** | Number of KOA patients’ appointments at specialist physicians^1^ |
| **Exclusion criteria** | - |
| **Quality objective** | *Aim: high value*  The QI represents the proportion of KOA patients for whom a referral precedes the billing contact with an orthopaedic surgeon.  A high proportion of KOA patients meeting the quality indicator’s requirements may indicate an increased coordination and continuity care. |
| **Rationale** | TJA is an effective therapy option to improve functionality and relieve pain and should therefore be performed after conservative therapy has proven ineffective. If symptoms persist despite conservative therapy, a referral should be made to an orthopaedic surgeon so that the necessity and chances of success of TJA can be assessed. |
| **Original wording** | If a patient is diagnosed with OA and has been referred to an orthopedic surgeon, then the waiting time from first referral should not exceed three months. [EUMUSC.net (2012)] |
| **Indicator variations** | - |
| **Dimension of quality** | Process |
| **Risk adjustment** | No risk adjustment |
| **Data source** | Routine data of statutory health insurances |
| **Literature references** | EUMUSC (2012) |

# LIST OF ABBREVIATIONS

**LIST OF ABBREVIATIONS**

CT Computer tomography

EBM Physicians’ fee schedule (Einheitlicher Bewertungsmaßstab)

IA Intra-articular

KOA Knee osteoarthritis

M2Q occurrence of a diagnosis over the course of a year, usually in two or more quarters

MRI Magnetic resonance imaging

PT Physical therapist

QI Quality indicator

SHI Statutory health insurance

TFA Subclaim type (Teilfallart)

TJA Total joint arthroplasty

Labels for diagnostic certainty:

A excluded diagnosis

G confirmed diagnosis

V suspected diagnosis

Z (asymptomatic) condition after the diagnosis in question

# REFERENCES

**REFERENCES**

**AWMF** (Arbeitsgemeinschaft der Wissenschaftlichen Medizinischen Fachgesellschaften) (Hrsg.), Deutsche Gesellschaft für Orthopädie und Unfallchirurgie e.V. (DGOU) (2021) Evidenz- und konsensbasierte Indikationskriterien zur Hüfttotalendoprothese bei Coxarthrose (EKIT-Hüfte). S3-Leitlinie der Deutschen Gesellschaft für Orthopädie und Unfallchirurgie e.V. (DGOU). <https://register.awmf.org/assets/guidelines/187-001l_S3_Indikationskriterien_H%C3%BCfttotalendoprothese_bei_Coxarthrose_2021-04.pdf>

**AWMF** (Arbeitsgemeinschaft der Wissenschaftlichen Medizinischen Fachgesellschaften) (Hrsg.), Deutsche Gesellschaft für Orthopädie und Unfallchirurgie e.V. (DGOU) (2019) S2k-Leitlinie Koxarthrose.

**AWMF** (Arbeitsgemeinschaft der Wissenschaftlichen Medizinischen Fachgesellschaften) (Hrsg.), Deutsche Gesellschaft für Orthopädie und Unfallchirurgie e.V. (DGOU) (2018) S2k-Leitlinie Gonarthrose.

**Doubova SV**, Perez-Cuevas R (2015) Quality of care for hip and knee osteoarthritis at family medicine clinics: lessons from Mexico. IJQHC 27(2):125-131. <https://doi.org/10.1093/intqhc/mzv003>

**ESCEO**, Bruyère O, Honvo G, Veronese N, Arden NK, Branco J, Curtis EM et al (2019) An updated algorithm recommendation for the management of knee osteoarthritis from the European Society for Clinical and Economic Aspects of Osteoporosis, Osteoarthritis and Musculoskeletal Diseases (ESCEO). Semin Arthritis Rheum 49(3):337-350. <https://doi.org/10.1016/j.semarthrit.2019.04.008>

**EULAR** (European Alliance of Associations for Rheumatology), Sakellariou G, Conaghan PG, Zhang W, Bijlsma JWJ, Boyesen P, D’Agostino MA et al (2017) EULAR recommendations for the use of imaging in the clinical management of peripheral joint osteoarthritis. Ann Rheum Dis 76(9):1484-1494. <https://doi.org/10.1136/annrheumdis-2016-210815>

**EUMUSC** (European Musculoskeletal Conditions Surveillance and Information Network) (2012) Health care quality indicators for OA. <http://www.eumusc.net/workpackages_wp6>. cfm. Accessed 10 Oct 2022.

**EULAR** (European Alliance of Associations for Rheumatology), Jordan KM, Arden NK, Doherty M, Bannwarth B, Bijlsma JW, Dieppe P et al (2003) EULAR Recommendations 2003: an evidence based approach to the management of knee osteoarthritis: Report of a Task Force of the Standing Committee for International Clinical Studies Including Therapeutic Trials (ESCISIT). Ann Rheum Dis 62(12):1145-1155. <https://doi.org/10.1136/ard.2003.011742>

**MacLean CH**, Pencharz JN, Saag KG (2007) Quality indicators for the care of osteoarthritis in vulnerable elders. JAGS 55 Suppl 2:S383-91. <https://doi.org/10.1111/j.1532-5415.2007.01346.x>

**NHG**, Belo JN, Bierma-Zeinstra SMA, Raaijmakers AJ, van der Wissel F, Opstelten W (2008) Nontraumatic Knee Complaints in Adults in General Practice. Huisarts en Wetenschap 51(5):229-240.

**NICE** (National Institute for Health and Care Excellence). National Institute for Health and Care Excellence (NICE) (2020) Osteoarthritis: care and management. <https://www.nice.org.uk/guidance/cg177>

**Saliba D**, Solomon D, Rubenstein L, Young R, Schnelle J, Roth C et al (2004) Quality indicators for the management of medical conditions in nursing home residents. JAMDA 5(5):297-309. <https://doi.org/10.1097/01.JAM.0000136960.25327.61>

**SFR** (French Society of Rheumatology), Sellam J, Courties A, Eymard F, Ferrero S, Latourte A, Ornetti P et al (2020) Recommendations of the French Society of Rheumatology on pharmacological treatment of knee osteoarthritis. Jt Bone Spine 87(6):548-555. <https://doi.org/10.1016/j.jbspin.2020.09.004>

**SIR** (Italian Society for Rheumatology), Ariani A, Manara M, Fioravanti A, Iannone F, Salaffi F, Ughi N, Prevete I et al (2019) The Italian Society for Rheumatology clinical practice guidelines for the diagnosis and management of knee, hip and hand osteoarthritis. Reumatismo 71(S1):5-21. <https://doi.org/10.4081/reumatismo.2019.1188>

**Smith KL**, Soriano TA, Boal J (2007) Brief communication: National quality-of-care standards in home-based primary care. Ann Intern Med 146(3):188-192. <https://doi.org/10.7326/0003-4819-146-3-200702060-00008>

**Steel N**, Melzer D, Shekelle PG, Wenger NS, Forsyth D, McWilliams BC (2004) Developing quality indicators for older adults: transfer from the USA to the UK is feasible. Qual Saf Health Care 13(4):260-264. <https://doi.org/10.1136/qhc.13.4.260>

**TLAR** (Turkish League Against Rheumatism), Tuncer T, Cay FH, Altan L, Gurer G, Kacar C, Ozcakir S et al (2018) 2017 update of the Turkish League Against Rheumatism (TLAR) evidence-based recommendations for the management of knee osteoarthritis. Rheumatol Int 38(8):1315-1331. <https://doi.org/10.1007/s00296-018-4044-y>

**Yabuki S**, Ip AKK, Tam CK, Murakami T, Ushida T, Wang JH et al (2019) Evidence-Based Recommendations on the Pharmacological Management of Osteoarthritis and Chronic Low Back Pain: An Asian Consensus. AJA 57(2):37-54. <https://doi.org/10.6859/aja.201906_57(2).0003>

Yeap SS, Amin SRA, Baharuddin H, Koh KC, Lee JK, Lee VKM et al (2021) A Malaysian Delphi consensus on managing knee osteoarthritis. BMC Musculoskelet Disord 22:514. <https://doi.org/10.1186/s12891-021-04381-8>

**Zhang Z**, Huang C, Jiang Q, Zheng Y, Liu Y, Liu S et al (2020) Guidelines for the diagnosis and treatment of osteoarthritis in China (2019 edition). Ann Transl Med 8(19):1213. <https://doi.org/10.21037/atm-20-4665>

**Zingmond DS**, Saliba D, Wilber KH, MacLean CH, Wenger NS (2009) Measuring the quality of care provided to dually enrolled Medicare and Medicaid beneficiaries living in nursing homes. Med Care 47(5):536-544. <https://doi.org/10.1097/MLR.0b013e318190cd8b>
